# Synthesis of pyrimido[1,6-*a*]quinoxalines via intermolecular trapping of thermally generated acyl(quinoxalin-2-yl)ketenes by Schiff bases

**DOI:** 10.3762/bjoc.14.147

**Published:** 2018-07-11

**Authors:** Svetlana O Kasatkina, Ekaterina E Stepanova, Maksim V Dmitriev, Ivan G Mokrushin, Andrey N Maslivets

**Affiliations:** 1 Department of Chemistry, Perm State University, ul. Bukireva 15, Perm 614990, Russian Federation

**Keywords:** acyl(quinoxalin-2-yl)ketenes, cycloaddition, pyrimido[1,6-*a*]quinoxalines, Schiff bases, thermolysis

## Abstract

Acyl(quinoxalin-2-yl)ketenes generated by thermal decarbonylation of 3-acylpyrrolo[1,2-*a*]quinoxaline-1,2,4(5*H*)-triones react regioselectively with Schiff bases under solvent-free conditions to form pyrimido[1,6-*a*]quinoxaline derivatives in good yields.

## Introduction

Quinoxaline is a 4-aza isostere of quinoline, which rarely occurs in structures of natural products. Its derivatives are gaining popularity in medicinal chemistry and pharmacology because many of them exhibit various biological activities [1–2].

**Figure 1:**
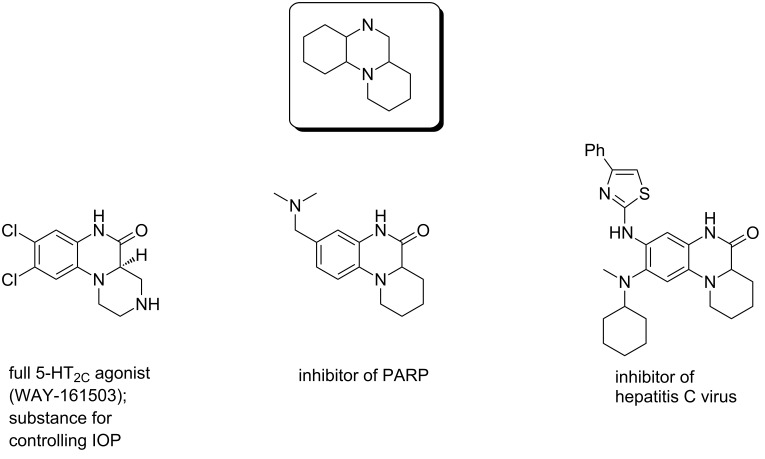
Quinoxaline-based 6/6/6-angularly fused scaffolds and respective examples of biologically active compounds.

Quinoxaline-based 6/6/6-angularly fused scaffolds (quinoxaline fused by a six-membered heterocycle at the [*a*]-side) are promising biologically active compounds. Recent research studies revealed that they can act as inhibitors of poly(ADP-ribose) polymerase (PARP) [3], inhibitors of hepatitis C virus [4], 5-HT_2C_ agonists [5–7], substances for controlling intraocular pressure (IOP) [8] etc. (Figure 1).

**Figure 2:**
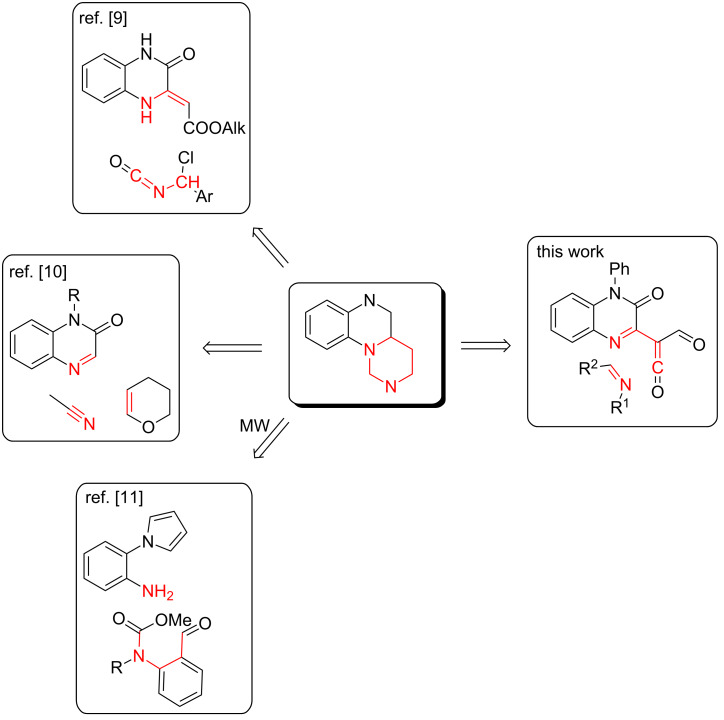
Synthetic routes towards the pyrimido[1,6-*a*]quinoxaline scaffold.

Pyrimido[1,6-*a*]quinoxalines are one of the most intriguing and unexplored structures representing isosteres of this scaffold. Only few synthetic procedures towards these compounds are described in the literature: heterocyclizations of α-chloroisocyanates with quinoxalin-2-ylideneacetates [9], multicomponent Mannich–Ritter transformations of quinoxalin-2(1*H*)-ones under the action of nitriles and 3,4-dihydro-2*H*-pyran [10] and a microwave-assisted cascade strategy via in situ-generated *N*-acyliminium ion precursors and amines [11] (Figure 2).

To develop a new synthetic approach towards pyrimido[1,6-*a*]quinoxalines we looked through the procedures to their closest analogues – pyrido[1,2-*a*]quinoxalines, the synthesis of which has been explored more frequently [3–4,12–46]. The analysis helped us to disclose a tempting but challenging methodology, which has the potential to be extended for the synthesis of the desired heterocyclic system, via intermolecular trapping of thermally generated acyl(quinoxalin-2-yl)ketenes [20–21,23–24,28–29,38] (Figure 2).

**Figure 3:**
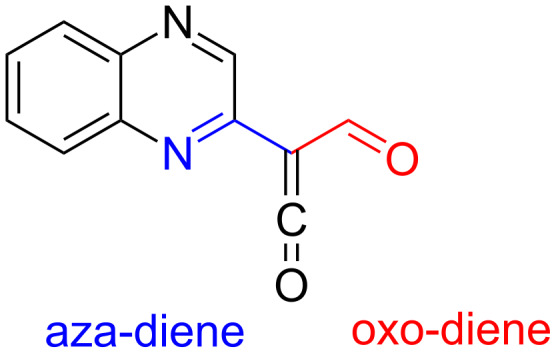
Acyl(quinoxalin-2-yl)ketene.

Syntheses utilizing acylketenes are of practical and theoretical interest due to the high reactivity of acylketenes and the structural diversity of the reaction products [47–54]. The introduction of the quinoxalin-2-yl substituent into acylketenes results in the formation of a peculiar system of conjugated double bonds, which can potentially act as either oxo-diene or aza-diene (Figure 3).

To the best of our knowledge, there is no example of the involvement of the aza-diene fragment of acyl(quinoxalin-2-yl)ketenes into intermolecular trapping by hetero-dienophiles published so far. In this article we report a synthetic protocol towards pyrimido[1,6-*a*]quinoxalines via the intermolecular trapping of acyl(quinoxalin-2-yl)ketenes by Schiff bases.

## Results and Discussion

**Scheme 1:**
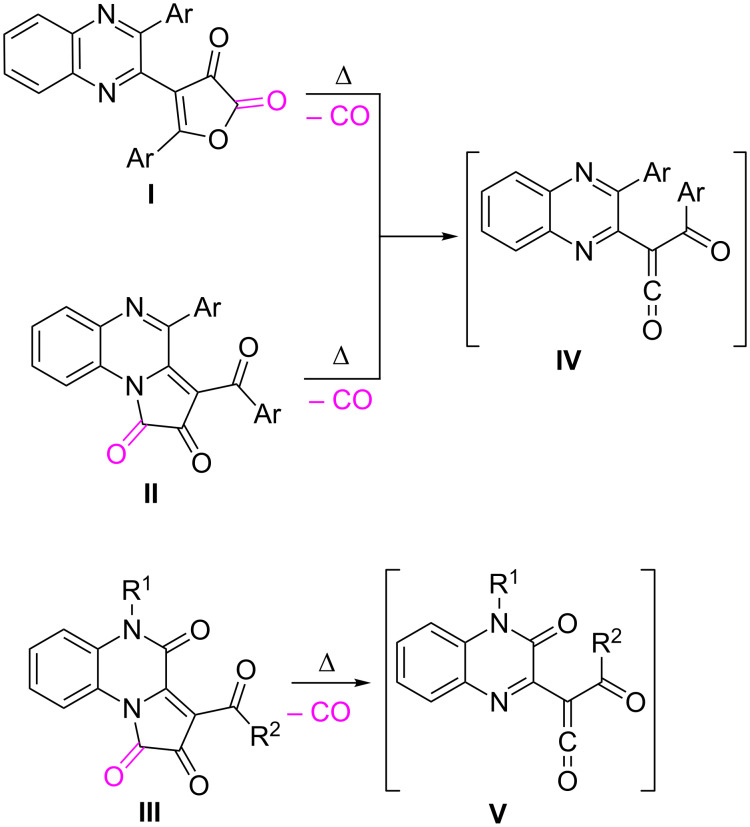
Thermolysis of five-membered 2,3-dioxoheterocycles resulting in acyl(quinoxalin-2-yl)ketenes.

The most convenient method for the generation of acyl(quinoxalin-2-yl)ketenes is the thermal decarbonylation (thermolysis) of five-membered 2,3-dioxoheterocycles having a quinoxaline fragment. Currently, three types of such precursors are known: 5-aryl-4-quinoxalin-2-ylfuran-2,3-diones **I** [21], 3-aroyl-4-arylpyrrolo[1,2-*a*]quinoxaline-1,2-diones **II** [55], and 3-acylpyrrolo[1,2-*a*]quinoxaline-1,2,4(5*H*)-triones **III** [23,56] (Scheme 1).

According to the literature data, precursors **I** and **II** are unsuitable for achieving the proposed goal as the generated ketene **IV** reacts only at its oxo-diene fragment in intermolecular trapping reactions with various dienophiles [57–62]. Under these circumstances precursors **III** generating ketenes **V** seemed to be the only suitable candidates for the development of a strategy towards pyrimido[1,6-*a*]quinoxalines.

**Figure 4:**
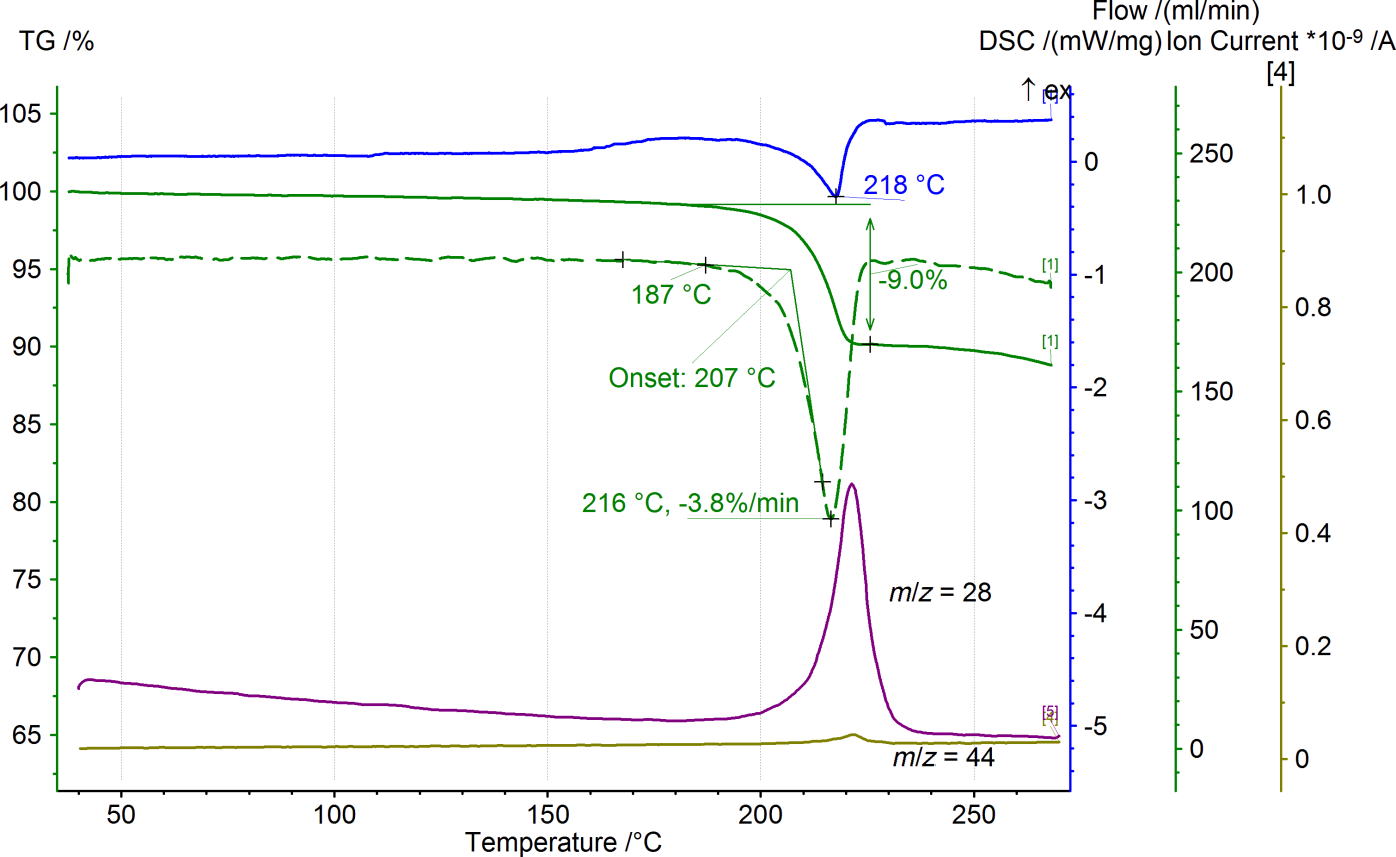
STA plot of thermolysis of PQT **1a**. Blue solid curve: DSC; green solid curve: TG; greed dashed curve: DTG; violet solid curve: MID (*m*/*z* = 28); brown solid curve: MID (*m*/*z* = 44); heating rate: 5 °C/min.

First, we studied the decarbonylation of precursors **III** – 3-acylpyrrolo[1,2-*a*]quinoxaline-1,2,4(5*H*)-triones (PQTs, **1a**–**h**) by simultaneous thermal analysis (STA, Table 1). According to the data obtained, PQTs **1a**–**h** underwent thermal decomposition with a mass loss accompanied by an endothermic effect and CO evolution (Figure 4). The values of the mass loss corresponded to the elimination of a CO molecule from a PQT.

**Table 1:** Thermal characteristics of decarbonylation of PQTs **1a**–**h**.

PQT	temp. of decarbonylation (°C)		
	
	onset	extrapolated onset	peak

**1a**	187	207	216
**1b**	174	209	220
**1c**	173	183	206
**1d**	148	174	183
**1e**	172	204	217
**1f**	179	198	212
**1g**	184	203	213
**1h**	171	197	206

Having taken into account the results of the thermal analysis, we examined the feasibility and conditions of the intermolecular reaction of the ketene generated from PQT **1a** with benzalaniline (**2a**). The reaction mixtures obtained were investigated by UPLC–MS and the results are summarized in Table 2.

**Table 2:** Intermolecular trapping of ketene generated from PQT **1a** by benzalaniline (**2a**)^a^.

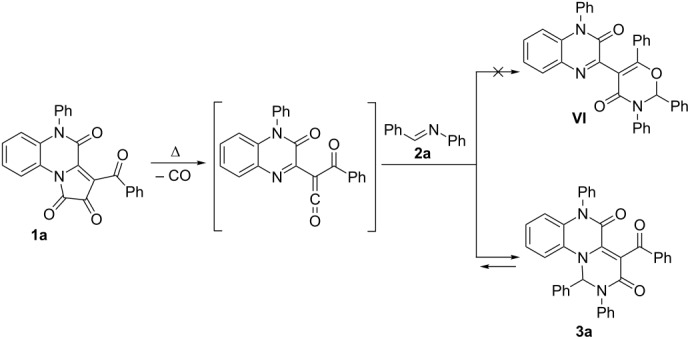			

entry	yield of **3a** (%)^b^	time (min)	temp. (°C)

1	65	5	190
2	50	15	190
3	57	5	200
4	30	60	200
5	80	3	175
6	85^c^	2	187

^a^Conditions: suspension of **1a** (1 mmol) and **2a** (1.1 mmol) in Dowtherm A (5 mL). ^b^Yields were determined by UPLC. ^c^Solvent-free reaction.

**Scheme 2:**
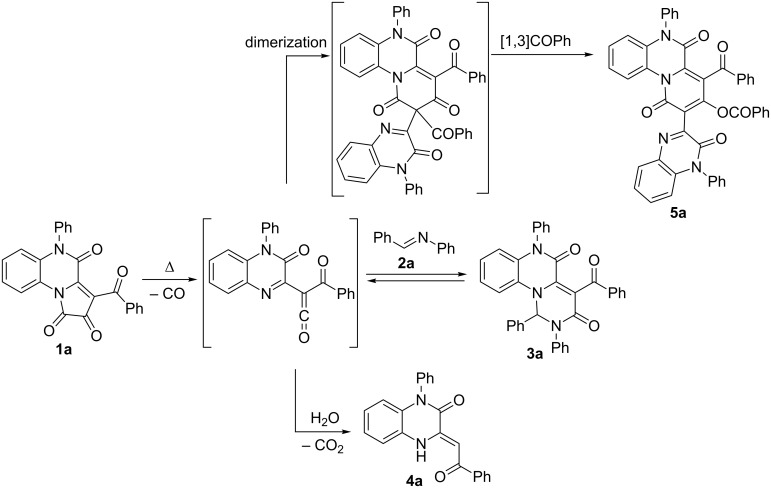
Side-reactions concurring with intermolecular trapping of ketene generated from PQT **1a** by benzalaniline (**2a**).

The reaction mixtures contained only three types of products, and we succeeded to identify each of them. The structures of the reaction products were elucidated as the desired pyrimido[1,6-*a*]quinoxaline **3a**, quinoxalinone **4a** [29] and pyrido[1,2-*a*]quinoxaline **5a** [29] (Scheme 2). Product **IV** of an alternative intermolecular trapping reaction (Table 1) was not detected.

The most likely way of the formation of quinoxalinone **4a** is hydration of the ketene with subsequent decarboxylation (Scheme 2); more careful drying the reaction vials and solvents easily reduced the amount of compound **4a**.

The formation of pyrido[1,2-*a*]quinoxaline **5a** can be explained by a concurrent process of ketene dimerization (Scheme 2) [29] in comparison to the intermolecular trapping of it by benzalaniline (**2a**). Since the yields of the target product **3a** decreased and the yields of compound **5a** increased at prolonged time of reaction, the formation of the target compounds deemed to be reversible.

Performing the reaction under solvent-free conditions at the onset decarbonylation temperature (Table 1) exceeded our expectations and gave the best yields of the target compound **3a** (Table 2, entry 6).

**Figure 5:**
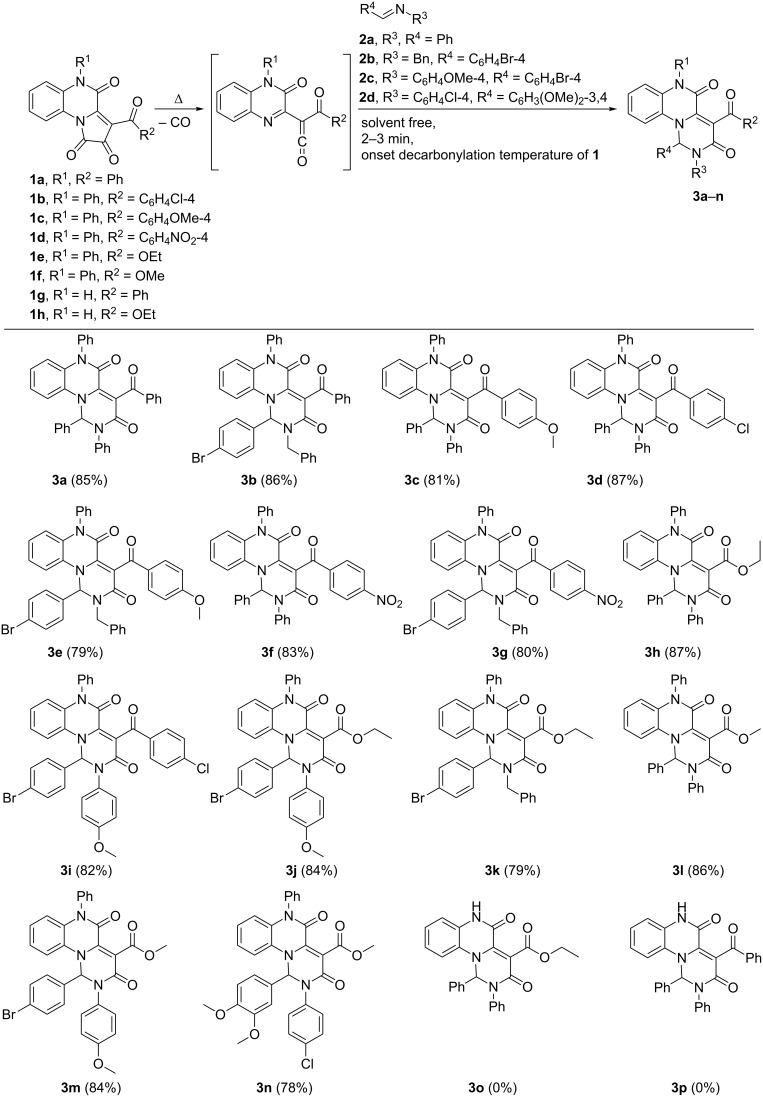
Scope of the intermolecular trapping of ketenes generated from PQTs **1a**–**h** by Schiff bases **2a**–**d** under solvent-free conditions.

Being inspired by the optimization results obtained, we examined the scope of the reaction applying the developed methodology with PQTs **1a**–**h** and Schiff bases **2a**–**d**. The results are shown in Figure 5.

Unfortunately, our attempts to involve Schiff bases synthesized from aliphatic aldehydes and ketones did not give any satisfactory results because of various nucleophilic side-reactions.

**Scheme 3:**
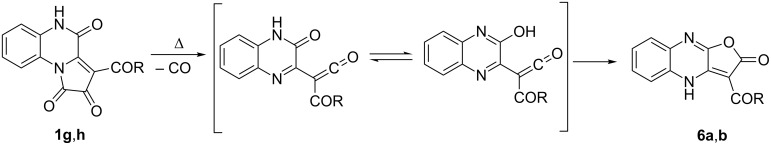
Formation of furoquinoxalines **6a**,**b** via intramolecular cyclization in ketenes generated from PQTs **1g**,**h**.

We found that the intermolecular trapping worked perfectly in case of N*^5^*-substituted PQTs **1a**–**f** and did not work at all with N*^5^*-unsubstituted PQT **1g** and **1h**. The failure to obtain products **3o** and **3p** from PQTs **1g** and **1h** can be explained by the occurrence of intramolecular cyclization in these ketenes resulting in the formation of furoquinoxalines **6a**,**b** [56,63–64] which were confirmed by UPLC–MS data as the sole products of the reaction (Scheme 3).

**Figure 6:**
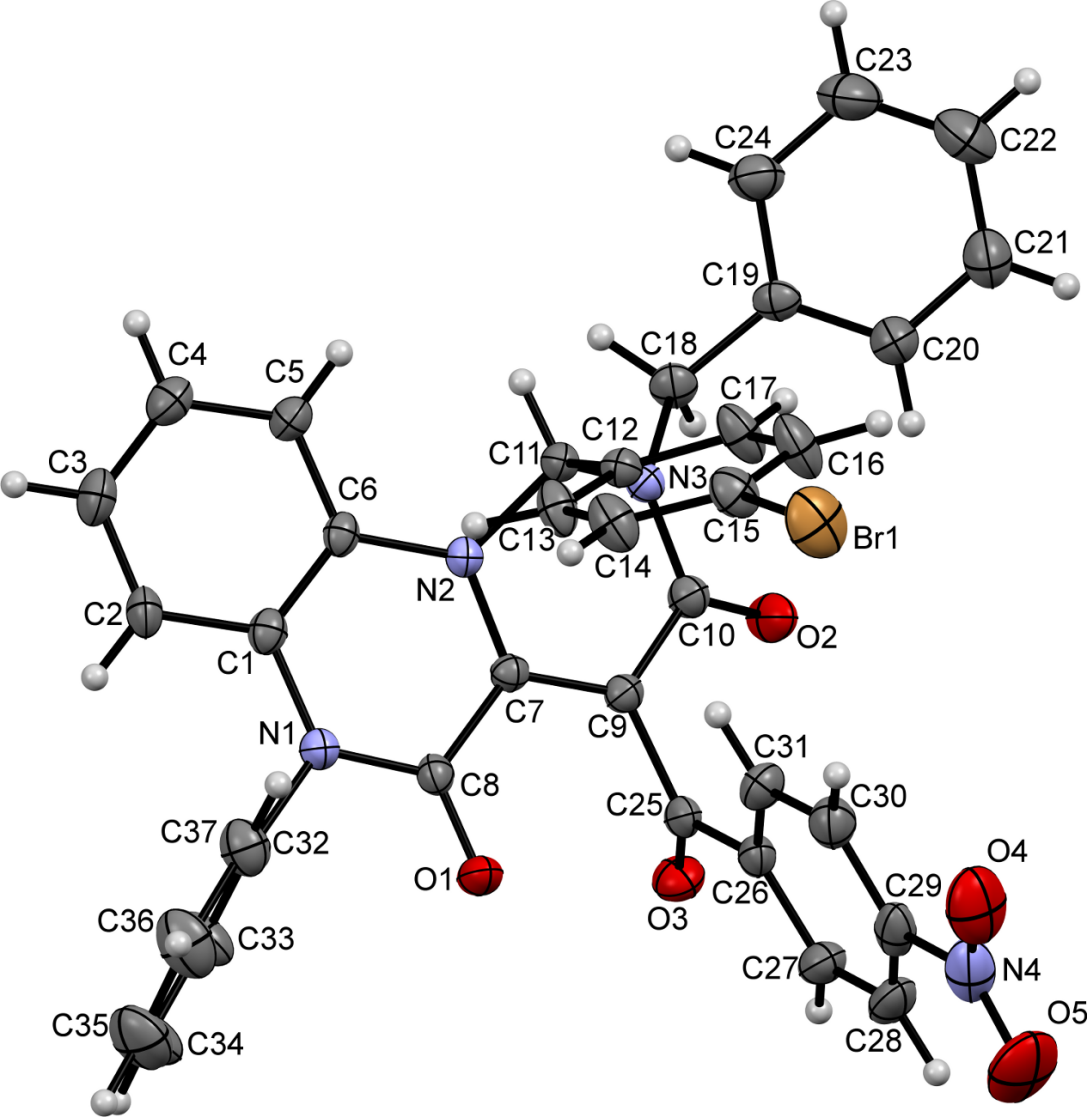
ORTEP drawing of compound **3g** (CCDC 1834011) showing thermal ellipsoids at the 30% probability level.

**Figure 7:**
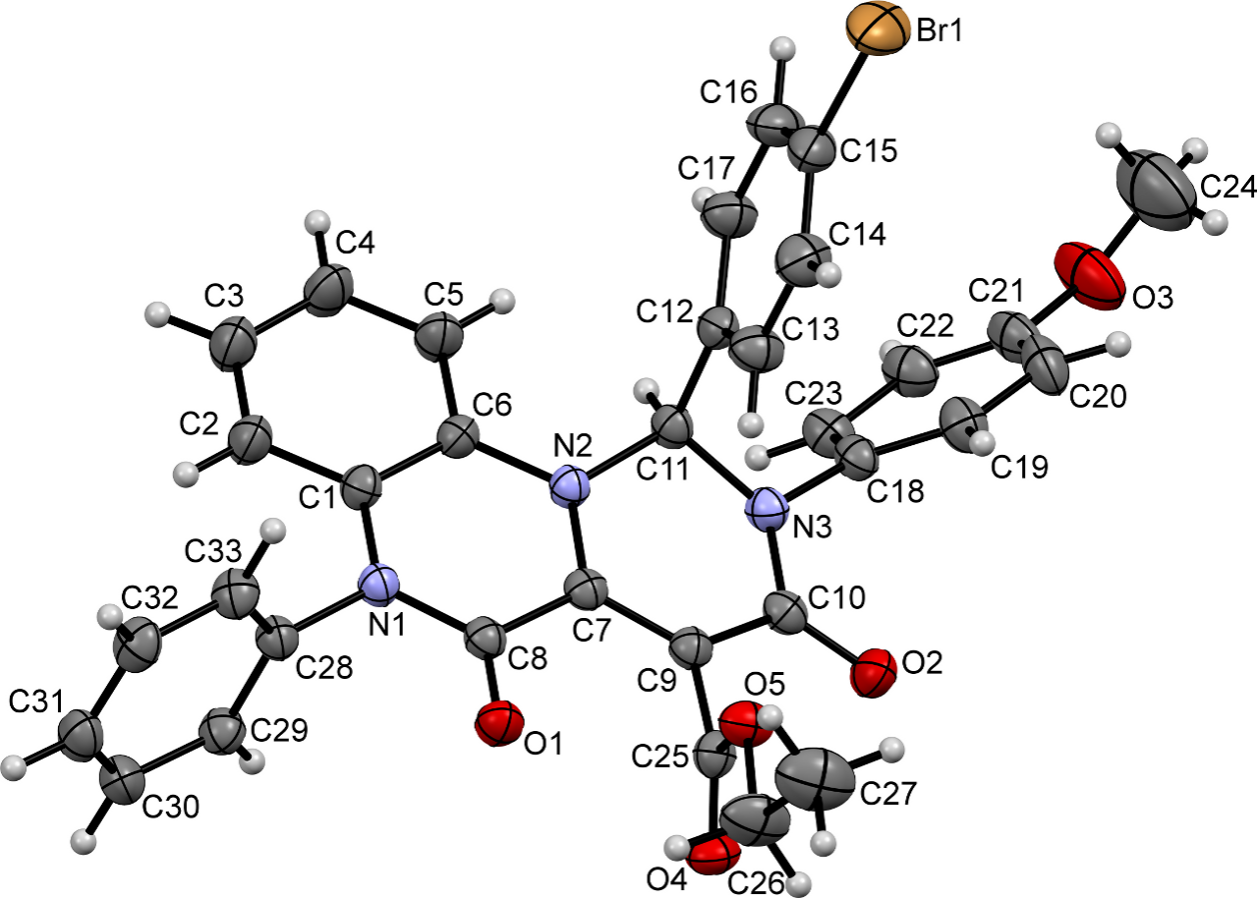
ORTEP drawing of compound **3j** (CCDC 1834012) showing thermal ellipsoids at the 30% probability level.

The formation of pyrimido[1,6-*a*]quinoxalines **3a**–**n** was unambiguously confirmed by the crystal structure of compounds **3g** and **3j** (CCDC 1834011, Figure 6; CCDC 1834012, Figure 7).

## Conclusion

We have developed a facile synthesis of pyrimido[1,6-*a*]quinoxaline derivatives via the intermolecular trapping of thermally generated acyl(quinoxalin-2-yl)ketenes by Schiff bases. The reaction proceeds under solvent-free conditions without any additives and catalysts. The elaborated method might be applicable to the syntheses of pharmaceutically important substances.

## Supporting Information

File 1 Experimental details, copies of ^1^H and ^13^C NMR spectra of pyrimido[1,6-*a*]quinoxalines **3a**–**n**, STA plots of PQT **1a**–**h** and X-ray crystal structure details of compounds **3g**,**j**.
