# Supplementary material for: Synthesis of pyrimido[1,6-a]quinoxalines via intermolecular trapping of thermally generated acyl(quinoxalin-2-yl)ketenes by Schiff bases
Source: Beilstein J Org Chem. 2018 Jul 11;14:1734–42. doi: 10.3762/bjoc.14.147 (PMC6071730; doi:10.3762/bjoc.14.147)
Supplement: File 1 — Experimental details, copies of 1H and 13C NMR spectra of pyrimido[1,6-a]quinoxalines 3a–n, STA plots of PQT 1a–h and X-ray crystal structure details of compounds 3g,j. [file Beilstein_J_Org_Chem-14-1734-s001.pdf]

Supporting information

for

Synthesis of pyrimido[1,6-*a*]quinoxalines via intermolecular trapping of thermally generated acyl(quinoxalin-2-yl)ketenes by Schiff bases

Svetlana O. Kasatkina, Ekaterina E. Stepanova*, Maksim V. Dmitriev, Ivan G. Mokrushin and Andrey N. Maslivets*

Address: Department of Chemistry, Perm State University, ul. Bukireva 15, Perm 614990, Russian Federation

Email: Ekaterina E. Stepanova* - caterina.stepanova@psu.ru; Andrey N. Maslivets* - koh2@psu.ru

* Corresponding author

Experimental details, copies of ^{1}H and ^{13}C NMR spectra of pyrimido[1,6-*a*]quinoxalines 3a–n, STA plots of PQT 1a–h and X-ray crystal structure details of compounds 3g,j

General

^{1}H and ^{13}C NMR spectra were acquired on a Bruker Avance-III spectrometer (400 and 100 MHz, respectively) in $CDCl_3$ or DMSO-d_6 using TMS or HMDSO as internal standards (^{1}H), or the solvent signal (^{13}C). IR spectra were recorded on a Perkin–Elmer Spectrum Two spectrometer from mulls in mineral oil. Melting points were measured on a Mettler Toledo MP90 apparatus. X-ray crystallography was performed on an Xcalibur Ruby diffractometer. Elemental analyses were carried out on a Vario MICRO Cube analyzer. The reaction conditions were optimized using UPLC (Waters ACQUITY UPLC I-Class system; Acquity UPLC BEH C18 column, grain size 1.7 µm; eluent acetonitrile–water, flow rate 0.6 mL/min; ACQUITY UPLC PDA eλ Detector; Xevo TQD mass detector, electrospray ionization, positive ion detection, ion source temperature 150 °C, capillary voltage 3500–4000 V, cone voltage 20–70 V, vaporizer temperature 150–300 °C). Simultaneous thermal analyses were done using a Netzsch STA 449 F1 Jupiter with temperature programs of 5 and 10 °C/min under an argon atmosphere (40 mL/min) between 45 and 450 °C. Starting 3-acylpyrrolo[1,2-*a*]quinoxaline-1,2,4(5*H*)-triones (PQT) **1a–h** were obtained according to reported procedures [1,2]. Other reagents and solvents were purchased from commercial vendors and were used as received.

Typical procedure to pyrimido[1,6-*a*]quinoxalines 3a–n:

A mixture of a PQT **1a–h** (1.0 mmol) and a Schiff base **2a–d** (1.1 mmol) was ground in a mortar, put into an oven-dried vial and pressed slightly. Then it was heated at onset decarbonylation temperature of the corresponding PQT (Table 1) for 2–3 min. The reaction mixture was cooled to room temperature and scrubbed with hexane (20 mL). The resulting precipitate was filtered off and recrystallized from toluene (for **3a-m**) or tetrachloromethane (for **3n**) to afford an appropriate pyrimido[1,6-*a*]quinoxalines **3a–n**.

4-Benzoyl-1,2,6-triphenyl-1,2-dihydro-3*H*-pyrimido[1,6-*a*]quinoxaline-3,5(6*H*)-dione (3a): A mixture of PQT **1a** (395 mg, 1.0 mmol) and Schiff base **2a** (200 mg, 1.1 mmol) was ground in a mortar, put into an oven-dried vial and pressed slightly, and then it

was heated at 187 °C for 2 min. The reaction mixture was cooled to room temperature and scrubbed with hexane (20 mL). The resulting precipitate was filtered off and recrystallized from toluene (15 mL) to afford pyrimido[1,6-*a*]quinoxaline **3a** as yellow solid, m.p. 257–259 °C. Yield 465 mg (0.85 mmol, 85%). ^{1}H NMR (400 MHz, $CDCl_3$) δ, ppm: 6.60 (m, 1H), 7.01 (m, 1H), 7.14 – 7.30 (m, 6H), 7.35 (m, 1H), 7.41 – 7.58 (m, 16H); ^{13}C NMR (100 MHz, $CDCl_3$) δ, ppm: 71.7, 112.2, 117.8, 123.2, 124.1, 124.8, 126.4, 126.7 (2C), 127.3, 128.0 (2C), 128.7 (2C), 129.0 (2C), 129.3 (2C), 129.3 (4C), 129.6, 129.7, 130.2 (2C), 132.3, 134.6, 135.6, 137.0, 137.8, 140.7, 155.4, 161.2, 191.5; IR (Mineral oil) ν, cm^{-1}: 1674, 1667, 1636; MS (ESI+): *m/z* $[M + H]^+$ calcd. for $C_{36}H_{26}N_3O_3$: 548.20; found: 548.23; Anal. calcd. for $C_{36}H_{25}N_3O_3$: C 78.96, H 4.60, N 7.67; found C 79.07, H 4.72, N 7.47.

4-Benzoyl-2-benzyl-1-(4-bromophenyl)-6-phenyl-1,2-dihydro-3*H*-pyrimido[1,6-*a*]quinoxaline-3,5(6*H*)-dione (3b): Yellow solid, m.p. 183–185 °C. Yield 550 mg (0.86 mmol, 86%). ^{1}H NMR (400 MHz, $CDCl_3$) δ, ppm: 4.03 (d, *J* 14.4 Hz, 1H), 5.82 (d, *J* 14.9 Hz, 1H), 6.50 (m, 1H), 6.60 (br.s, 1H), 6.73 (s, 1H), 6.90 (m, 1H), 6.98 (m, 1H), 7.18 – 7.31 (m, 6H), 7.40 – 7.59 (m, 12H); ^{13}C NMR (100 MHz, $CDCl_3$) δ, ppm: 48.4, 67.2, 111.7, 117.6, 123.0, 124.0, 124.5, 126.9, 128.1 (4C), 128.4, 128.6 (2C), 128.6, 128.7 (2C), 129.0 (2C), 129.3 (2C), 129.5, 130.2 (2C), 132.5, 132.5 (2C), 134.6, 135.5, 135.6, 137.8, 155.5, 161.9, 191.5; IR (Mineral oil) ν, cm^{-1}: 1674, 1629; MS (ESI+): *m/z* $[M + H]^+$ calcd. for $C_{37}H_{27}BrN_3O_3$: 640.12, 642.12; found: 640.14, 642.12; Anal. calcd. for $C_{37}H_{26}BrN_3O_3$: C 69.38, H 4.09, N 6.56; found C 69.49, H 4.22, N 6.57.

4-(4-Methoxybenzoyl)-1,2,6-triphenyl-1,2-dihydro-3*H*-pyrimido[1,6-*a*]quinoxaline-3,5(6*H*)-dione (3c): Yellow solid, m.p. 225–227 °C. Yield 467 mg (0.81 mmol, 81%). ^{1}H NMR (400 MHz, $CDCl_3$) δ, ppm: 3.76 (s, 3H), 6.60 (m, 3H), 7.00 (m, 1H), 7.21 – 7.57 (m, 20H); ^{13}C NMR (100 MHz, $CDCl_3$) δ, ppm: 55.3, 71.5 (br), 112.1, 113.4 (2C), 117.7, 123.2, 124.0 (2C), 124.7, 126.3, 126.7 (2C), 127.4, 128.7 (2C), 129.3, 129.3 (4C), 129.3, 129.6, 130.2 (2C), 131.3 (2C), 131.3, 134.3, 135.7, 137.4, 140.8, 155.4, 161.3, 163.1, 190.2; IR (Mineral oil) ν, cm^{-1}: 1679, 1673, 1634; MS (ESI+): *m/z* $[M + H]^+$ calcd. for $C_{37}H_{28}N_3O_4$: 578.21; found: 578.19; Anal. calcd. for $C_{37}H_{27}N_3O_4$: C 76.93, H 4.71, N 7.27; found C 76.95, H 4.78, N 7.23.

4-(4-Chlorobenzoyl)-1,2,6-triphenyl-1,2-dihydro-3*H*-pyrimido[1,6-*a*]quinoxaline-3,5(6*H*)-dione (3d): Yellow solid, m.p. 251–253 °C. Yield 505 mg (0.87 mmol, 87%). ^{1}H NMR (400 MHz, $CDCl_3$) δ, ppm: 6.61 (m, 1H), 7.03 (m, 1H), 7.12 (m, 2H), 7.23 – 7.56 (m, 20H); ^{13}C NMR (100 MHz, $CDCl_3$) δ, ppm: 71.8, 112.3, 117.8, 123.4, 124.1 (2C), 124.9, 126.5, 126.7 (2C), 127.1, 128.4 (2C), 128.6 (2C), 129.3 (2C), 129.4 (2C), 129.5, 129.5, 129.8, 130.3 (4C), 134.8, 135.4, 136.4, 137.0, 137.7, 140.6, 155.4, 161.1, 190.2; IR (Mineral oil) ν, cm^{-1}: 1672, 1639; MS (ESI+): *m/z* $[M + H]^+$ calcd. for $C_{36}H_{25}ClN_3O_3$: 582.16; found: 582.13; Anal. calcd. for $C_{36}H_{24}ClN_3O_3$: C 74.29, H 4.16, N 7.22; found C 74.33, H 4.32, N 7.27.

2-Benzyl-1-(4-bromophenyl)-4-(4-methoxybenzoyl)-6-phenyl-1,2-dihydro-3*H*-pyrimido[1,6-*a*]quinoxaline-3,5(6*H*)-dione (3e): Yellow solid, m.p. 243–245 °C. Yield 529 mg (0.79 mmol, 79%). ^{1}H NMR (400 MHz, $CDCl_3$) δ, ppm: 3.80 (s, 3H), 4.06 (m, 1H), 5.83 (m, 1H), 6.49 (m, 2H), 6.71 (m, 3H), 6.86 – 6.96 (m, 2H), 7.17 – 7.59 (m, 15H); ^{13}C NMR (400 MHz, $CDCl_3$) δ, ppm: 48.9), 55.4, 66.8, 111.6, 113.5 (2C), 117.5, 122.9, 123.8, 124.4, 127.1, 128.1 (2C), 128.4, 128.8 (4C), 129.0, 129.3, 129.3 (2C), 129.6, 130.2 (2C), 131.2 (3C), 132.5 (2C), 134.4, 135.6, 135.6, 136.3, 155.6, 161.9, 163.2, 190.0; IR (Mineral oil) ν, cm^{-1}: 1671, 1660, 1628; MS (ESI+): *m/z* $[M + H]^+$ calcd. for $C_{38}H_{29}BrN_3O_4$: 670.13, 672.13; found: 670.17, 673.15; Anal. calcd. for $C_{38}H_{28}BrN_3O_4$: C 68.06, H 4.21, N 6.27; found C 68.05, H 4.32, N 6.33.

4-(4-Nitrobenzoyl)-1,2,6-triphenyl-1,2-dihydro-3*H*-pyrimido[1,6-*a*]quinoxaline-3,5(6*H*)-dione (3f): Yellow solid, m.p. 169–170 °C. Yield 492 mg (0.83 mmol, 83%). ^{1}H NMR (400 MHz, DMSO-d_6) δ, ppm: 6.46 (m, 1H), 7.10 (m, 1H), 7.27 – 7.37 (m, 4H), 7.44 – 7.56 (m, 13H), 7.80 (br.s, 1H), 7.90 (s, 1H), 8.09 – 8.16 (m, 3H); ^{13}C NMR (100 MHz, DMSO-d_6) δ, ppm: 71.3, 109.0, 113.9, 116.6, 123.2 (2C), 123.5, 124.5, 125.1 (2C), 126.1, 126.4 (2C), 126.5, 128.8 (2C), 129.0 (2C), 129.2 (2C), 129.2, 129.3 (2C), 130.0 (2C), 135.6, 135.8, 137.0, 139.9, 142.6, 149.2, 154.9, 161.1, 189.8; IR (Mineral oil) ν, cm^{-1}: 1681, 1643; MS (ESI+): *m/z* $[M + H]^+$ calcd. for $C_{36}H_{25}N_4O_5$: 593.18; found: 593.16; Anal. calcd. for $C_{36}H_{24}N_4O_5$: C 72.96, H 4.08, N 9.45; found C 72.87, H 4.02, N 9.67.

2-Benzyl-1-(4-bromophenyl)-4-(4-nitrobenzoyl)-6-phenyl-1,2-dihydro-3*H*-pyrimido[1,6-*a*]quinoxaline-3,5(6*H*)-dione (3g): Yellow solid, m.p. 222–224 °C. Yield 393 mg (0.80 mmol, 80%). ^{1}H NMR (400 MHz, $CDCl_3$) δ, ppm: 4.04 (d, *J* 14.9 Hz, 1H), 5.79 (d, *J* 14.9 Hz, 1H), 6.53 (m, 1H), 6.67 (m, 1H), 6.75 (s, 1H), 6.94 (m, 1H), 7.04 (m, 1H), 7.17 (br.s, 2H), 7.29 (m, 2H), 7.40 – 7.49 (m, 8H), 7.60 (m, 2H), 7.76 (br.s, 2H), 8.12 (m, 2H); ^{13}C NMR (100 MHz, $CDCl_3$) δ, ppm: 48.3, 67.6, 112.0, 117.7, 123.5, 123.5 (2C), 124.8, 126.4, 128.1 (2C), 128.4 (2C), 128.6, 128.7 (2C), 129.0, 129.4, 129.4 (2C), 129.6, 129.7 (2C), 130.3 (2C), 132.7 (2C), 135.0, 135.2, 135.3, 142.5, 149.9, 155.4, 161.8, 189.8; IR (Mineral oil) ν, cm^{-1}: 1690, 1660, 1634; MS (ESI+): *m/z* $[M + H]^+$ calcd. for $C_{37}H_{26}BrN_4O_5$: 685.11, 687.11; found: 685.17, 687.12; Anal. calcd. for $C_{37}H_{25}BrN_4O_5$: C 64.83, H 3.68, N 8.17; found C 64.99, H 3.52, N 8.57.

Ethyl 3,5-dioxo-1,2,6-triphenyl-2,3,5,6-tetrahydro-1*H*-pyrimido[1,6-*a*]quinoxaline-4-carboxylate (3h): Yellow solid, m.p. 229–231 °C. Yield 449 mg (0.87 mmol, 87%). ^{1}H NMR (400 MHz, $CDCl_3$) δ, ppm: 1.29 (t, *J* 7.1 Hz, 3H), 4.31 (m, 2H), 6.55 (m, 1H), 6.97 (m, 1H), 7.17 – 7.30 (m, 5H), 7.37 – 7.42 (m, 8H), 7.47 – 7.60 (m, 5H); ^{13}C NMR (100 MHz, $CDCl_3$) δ, ppm: 14.0, 61.6, 72.4, 108.8, 112.4, 117.6, 123.2, 124.6, 125.1 (2C), 126.5 (2C), 126.6, 126.8, 128.7 (2C), 129.2 (2C), 129.3 (2C), 129.5, 129.6, 129.8, 130.3 (2C), 134.2, 135.7, 136.0, 140.4, 154.9, 160.8, 165.0; IR (Mineral oil) ν, cm^{-1}: 1732, 1682, 1644; MS (ESI+): *m/z* $[M + H]^+$ calcd. for $C_{32}H_{26}N_3O_4$: 516.19; found: 516.21; Anal. calcd. for $C_{32}H_{25}N_3O_4$: C 74.55, H 4.89, N 8.15; found C 74.47, H 4.72, N 8.02.

1-(4-Bromophenyl)-4-(4-chlorobenzoyl)-2-(4-methoxyphenyl)-6-phenyl-1,2-dihydro-3*H*-pyrimido[1,6-*a*]quinoxaline-3,5(6*H*)-dione (3i): Yellow solid, m.p. 243–244 °C. Yield 566 mg (0.82 mmol, 82%). ^{1}H NMR (400 MHz, $CDCl_3$) δ, ppm: 3.83 (s, 3H), 6.60 (m, 1H), 6.94 (m, 2H), 7.02 (m, 1H), 7.21 – 7.63 (m, 18H); ^{13}C NMR (100 MHz, $CDCl_3$) δ, ppm: 55.6, 72.1 (br), 112.2, 114.7 (2C), 115.0, 117.9, 123.5, 124.2, 124.9, 126.0 (br), 126.8, 128.2, 128.4 (2C), 128.4 (2C), 128.6 (4C), 129.0, 129.5, 129.6, 130.3 (2C), 130.3 (2C), 132.6 (2C), 133.1, 134.6, 135.4, 136.4, 138.8, 155.3, 161.0, 190.2; IR (Mineral oil) ν, cm^{-1}: 1679, 1666, 1641; MS (ESI+): *m/z* $[M + H]^+$

calcd. for $C_{37}H_{26}BrClN_3O_4$: 690.08, 692.08; found: 690.12, 692.12; Anal. calcd. for $C_{37}H_{25}BrClN_3O_4$: C 64.32, H 3.65, N 6.08; found C 64.35, H 3.61, N 6.07.

Ethyl 1-(4-bromophenyl)-2-(4-methoxyphenyl)-3,5-dioxo-6-phenyl-2,3,5,6-tetrahydro-1*H*-pyrimido[1,6-*a*]quinoxaline-4-carboxylate (3j): Yellow solid, m.p. 225–227 °C. Yield 449 mg (0.84 mmol, 84%). ^{1}H NMR (400 MHz, $CDCl_3$) δ, ppm: 1.30 (t, *J* 7.2 Hz, 3H), 3.82 (s, 3H), 4.32 (m, 2H), 6.55 (m, 1H), 6.90 – 6.99 (m, 3H), 7.13 (s, 1H), 7.18 (m, 1H), 7.22 – 7.30 (m, 4H), 7.34 – 7.37 (m, 3H), 7.50 – 7.59 (m, 5H); ^{13}C NMR (100 MHz, $CDCl_3$) δ, ppm: 14.0, 55.5, 62.0, 72.5, 108.6, 112.2, 114.7 (2C), 117.7, 123.3, 124.1, 124.6, 126.9 (2C), 128.2 (2C), 128.2, 128.7 (2C), 129.5, 129.6, 130.3, 132.4 (2C), 132.9 (2C), 134.2, 135.2, 135.7, 154.8, 158.5, 160.6, 164.9; IR (Mineral oil) ν, cm^{-1}: 1737, 1688, 1650; MS (ESI+): *m/z* $[M + H]^+$ calcd. for $C_{33}H_{27}BrN_3O_5$: 624.11, 626.11; found: 624.12, 626.13; Anal. calcd. for $C_{33}H_{26}BrN_3O_5$: C 63.47, H 4.20, N 6.73; found C 63.49, H 4.32, N 6.62.

Ethyl 2-benzyl-1-(4-bromophenyl)-3,5-dioxo-6-phenyl-2,3,5,6-tetrahydro-1*H*-pyrimido[1,6-*a*]quinoxaline-4-carboxylate (3k): Yellow solid, m.p. 169–171 °C. Yield 481 mg (0.79 mmol, 79%). ^{1}H NMR (400 MHz, $CDCl_3$) δ, ppm: 1.33 (t, *J* 7.2 Hz, 3H), 3.88 (d, *J* 15,2 Hz, 1H), 4.35 (m, 2H), 5.78 (d, *J* 15,2 Hz, 1H), 6.46 (m, 1H), 6.61 (s, 1H), 6.66 (m, 1H), 6.87 (m, 1H), 6.98 (m, 1H), 7.24 – 7.29 (m, 4H), 7.36 – 7.42 (m, 5H), 7.49 – 7.56 (m, 5H); ^{13}C NMR (100 MHz, $CDCl_3$) δ, ppm: 14.0, 47.6, 61.7, 67.6, 107.9, 111.8, 117.5, 123.0, 124.1, 124.4, 126.3, 128.1 (2C), 128.2 (2C), 128.5 (2C), 128.7, 129.2 (2C), 129.5, 129.6, 130.3 (2C), 132.5 (2C), 134.3, 135.4, 135.7, 137.9, 155.0, 161.4, 165.0; IR (Mineral oil) ν, cm^{-1}: 1725, 1680, 1639; MS (ESI+): *m/z* $[M + H]^+$ calcd. for $C_{33}H_{27}BrN_3O_4$: 608.12, 610.12; found: 608.10, 610.11; Anal. calcd. for $C_{33}H_{26}BrN_3O_4$: C 65.14, H 4.31, N 6.91; found C 65.22, H 4.32, N 7.02.

Methyl 3,5-dioxo-1,2,6-triphenyl-2,3,5,6-tetrahydro-1*H*-pyrimido[1,6-*a*]quinoxaline-4-carboxylate (3l): Yellow solid, m.p. 245–247 °C. Yield 431 mg (0.86 mmol, 86%). ^{1}H NMR (400 MHz, $CDCl_3$) δ, ppm: 3.83 (s, 3H), 6.56 (m, 1H), 6.99 (m, 1H), 7.17 – 7.25 (m, 2H), 7.27 – 7.31 (m, 3H), 7.37 – 7.48 (m, 10H), 7.50 – 7.60 (m, 3H); ^{13}C NMR (100 MHz, $CDCl_3$) δ, ppm: 52.7, 72.4, 108.5, 112.5, 117.6,

123.3, 124.7, 125.0 (2C), 126.5 (2C), 126.8, 128.2, 128.7 (2C), 129.3 (2C), 129.3 (2C), 129.5, 129.6, 129.8, 130.4 (2C), 134.4, 135.7, 136.0, 140.4, 155.0, 160.8, 165.5; IR (Mineral oil) ν, cm^{-1}: 1726, 1678, 1651; MS (ESI+): *m/z* $[M + H]^+$ calcd. for $C_{31}H_{24}N_3O_4$: 502.18; found: 502.15; Anal. calcd. for $C_{31}H_{23}N_3O_4$: C 74.24, H 4.62, N 8.38; found C 74.35, H 4.37, N 8.22.

Methyl 1-(4-bromophenyl)-2-(4-methoxyphenyl)-3,5-dioxo-6-phenyl-2,3,5,6-tetrahydro-1*H*-pyrimido[1,6-*a*]quinoxaline-4-carboxylate (3m): Yellow solid, m.p. 245–246 °C. Yield 512 mg (0.84 mmol, 84%). ^{1}H NMR (400 MHz, $CDCl_3$) δ, ppm: 3.82 (s, 3H), 3.83 (s, 3H), 6.55 (m, 1H), 6.92 (m, 2H), 6.98 (m, 1H), 7.14 (s, 1H), 7.17 – 7.30 (m, 5H), 7.33 – 7.37 (m, 3H), 7.50 – 7.60 (m, 5H); ^{13}C NMR (100 MHz, $CDCl_3$) δ, ppm: 52.7, 55.5, 72.5, 108.2, 112.3, 114.7 (2C), 117.7, 123.4, 124.2, 124.7, 126.3, 126.8 (2C), 128.2 (2C), 128.5 (2C), 128.7, 129.6, 130.4 (2C), 132.5 (2C), 132.9, 134.3, 135.1, 135.6, 154.8, 158.5, 160.6, 165.4; IR (Mineral oil) ν, cm^{-1}: 1739, 1677, 1641; MS (ESI+): *m/z* $[M + H]^+$ calcd. for $C_{32}H_{25}BrN_3O_5$: 610.10, 612.10; found: 610.13, 612.11; Anal. calcd. for $C_{32}H_{24}BrN_3O_5$: C 62.96, H 3.96, N 6.88; found C 62.99, H 4.07, N 6.81.

Methyl 2-(4-chlorophenyl)-1-(3,4-dimethoxyphenyl)-3,5-dioxo-6-phenyl-2,3,5,6-tetrahydro-1*H*-pyrimido[1,6-*a*]quinoxaline-4-carboxylate (3n): Yellow solid, m.p. 184–185 °C. Yield 464 mg (0.78 mmol, 78%). ^{1}H NMR (400 MHz, $CDCl_3$) δ, ppm: 3.81 (s, 3H), 3.83 (s, 3H), 3.88 (s, 3H), 6.55 (m, 1H), 6.84 (m, 1H), 6.95 – 7.00 (m, 2H), 7.08 (m, 1H), 7.14 (s, 1H), 7.18 – 7.30 (m, 5H), 7.36 (m, 2H), 7.43 (m, 1H), 7.50 – 7.59 (m, 3H); ^{13}C NMR (100 MHz, $CDCl_3$) δ, ppm: 52.7, 56.0, 56.1, 72.6, 107.7, 109.7, 111.6, 112.5, 117.7, 119.2, 123.4, 124.6, 126.1, 126.6 (4C), 128.1, 128.6 (2C), 129.4 (4C), 129.6, 129.6, 130.4 (2C), 132.5, 134.5, 135.6, 138.7, 149.83, 150.6, 154.7, 160.8, 165.5; IR (Mineral oil) ν, cm^{-1}: 1743, 1683, 1638; MS (ESI+): *m/z* $[M + H]^+$ calcd. for $C_{33}H_{27}ClN_3O_6$: 596.16; found: 596.18; Anal. calcd. for $C_{33}H_{26}ClN_3O_6$: C 66.50, H 4.40, N 7.05; found C 66.46, H 4.35, N 6.91.

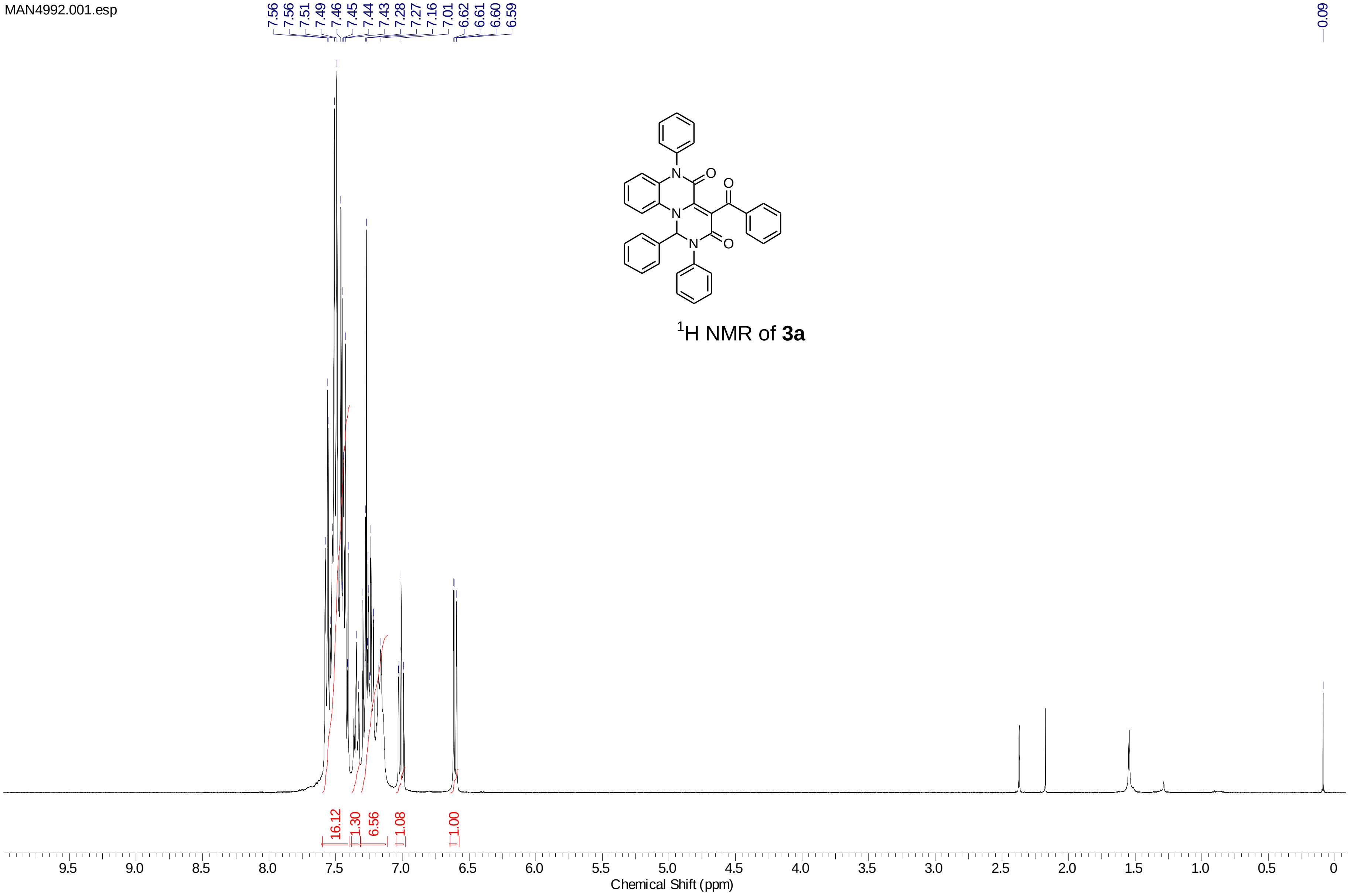
MAN4992.001.esp
7.56 7.56 7.51 7.49 7.46 7.45 7.44 7.43 7.28 7.27 7.16 7.01 6.62 6.61 6.60 6.59
0.09
N
O
O
N
N
O
16.12 1.30 6.56 1.08 1.00
9.5 9.0 8.5 8.0 7.5 7.0 6.5 6.0 5.5 5.0 4.5 4.0 3.5 3.0 2.5 2.0 1.5 1.0 0.5 0
Chemical Shift (ppm)

^{1}H NMR of **3a**

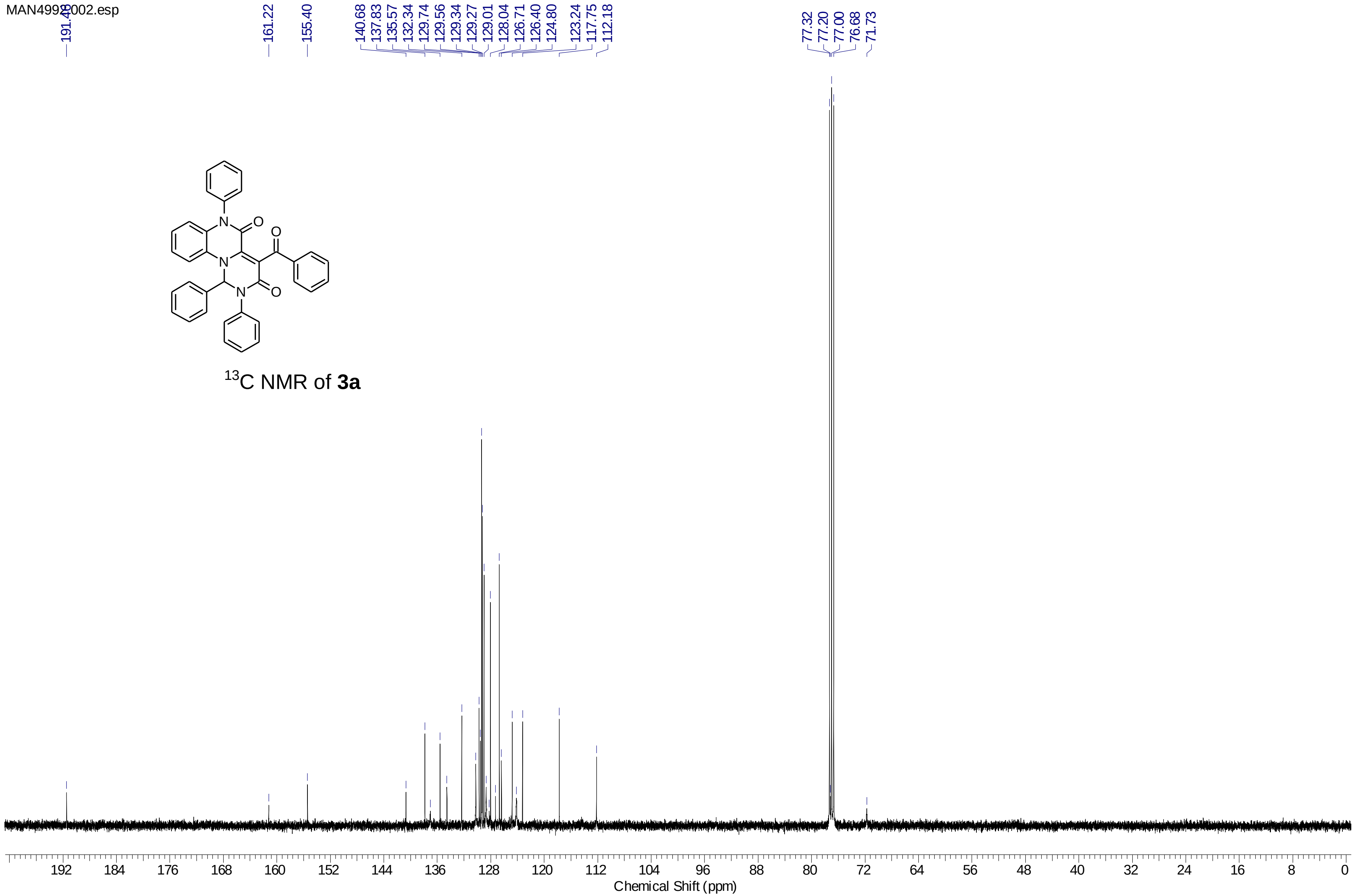
MAN4992002.esp
191.40
161.22
155.40
140.68
137.83
135.57
132.34
129.74
129.56
129.34
129.27
129.01
128.04
126.71
126.40
124.80
123.24
117.75
112.18
77.32
77.20
77.00
76.68
71.73
192
184
176
168
160
152
144
136
128
120
112
104
96
88
80
72
64
56
48
40
32
24
16
8
0
Chemical Shift (ppm)

^{13}C NMR of **3a**

MAN5108.001.esp

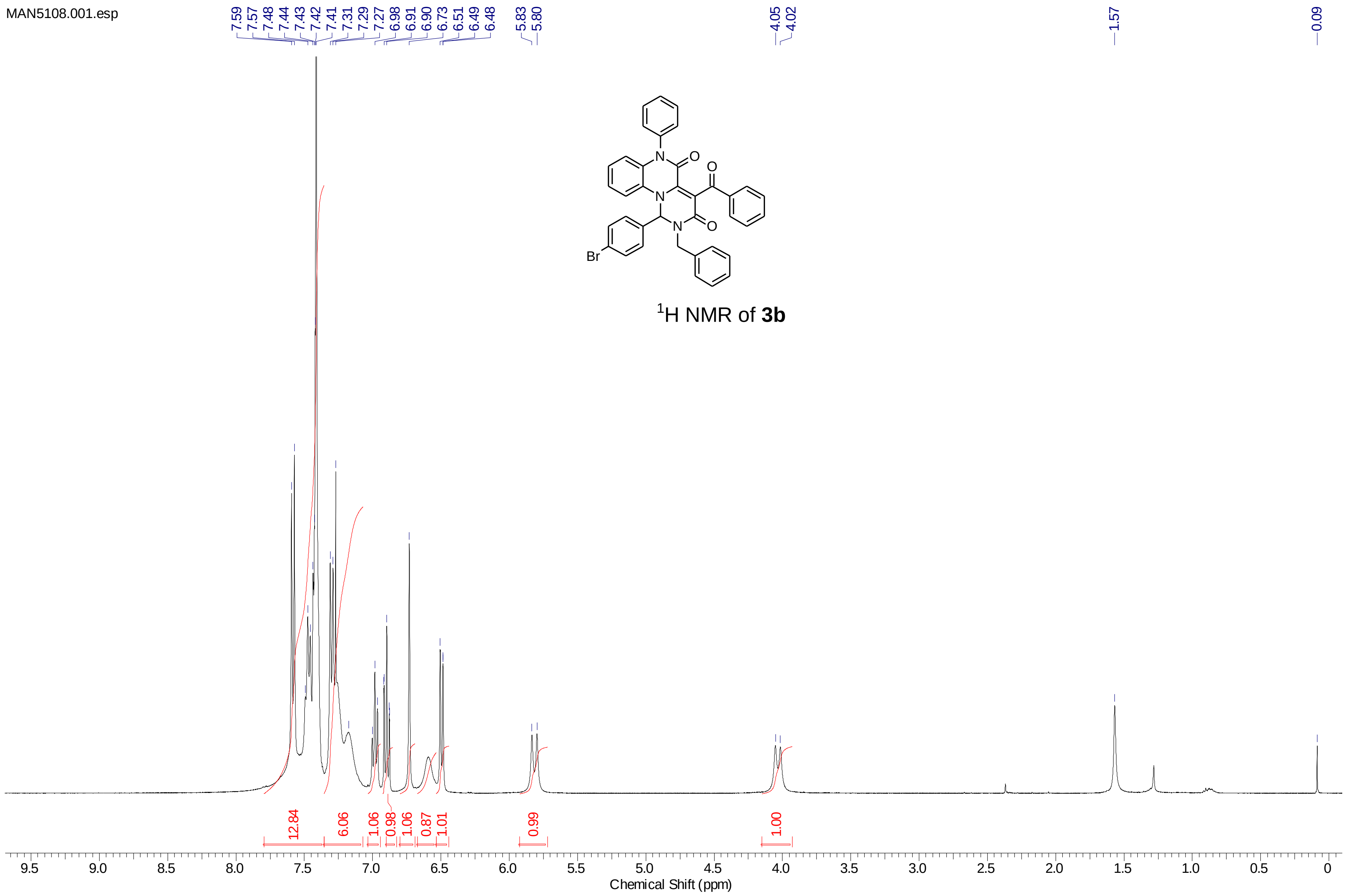

^{1}H NMR of **3b**

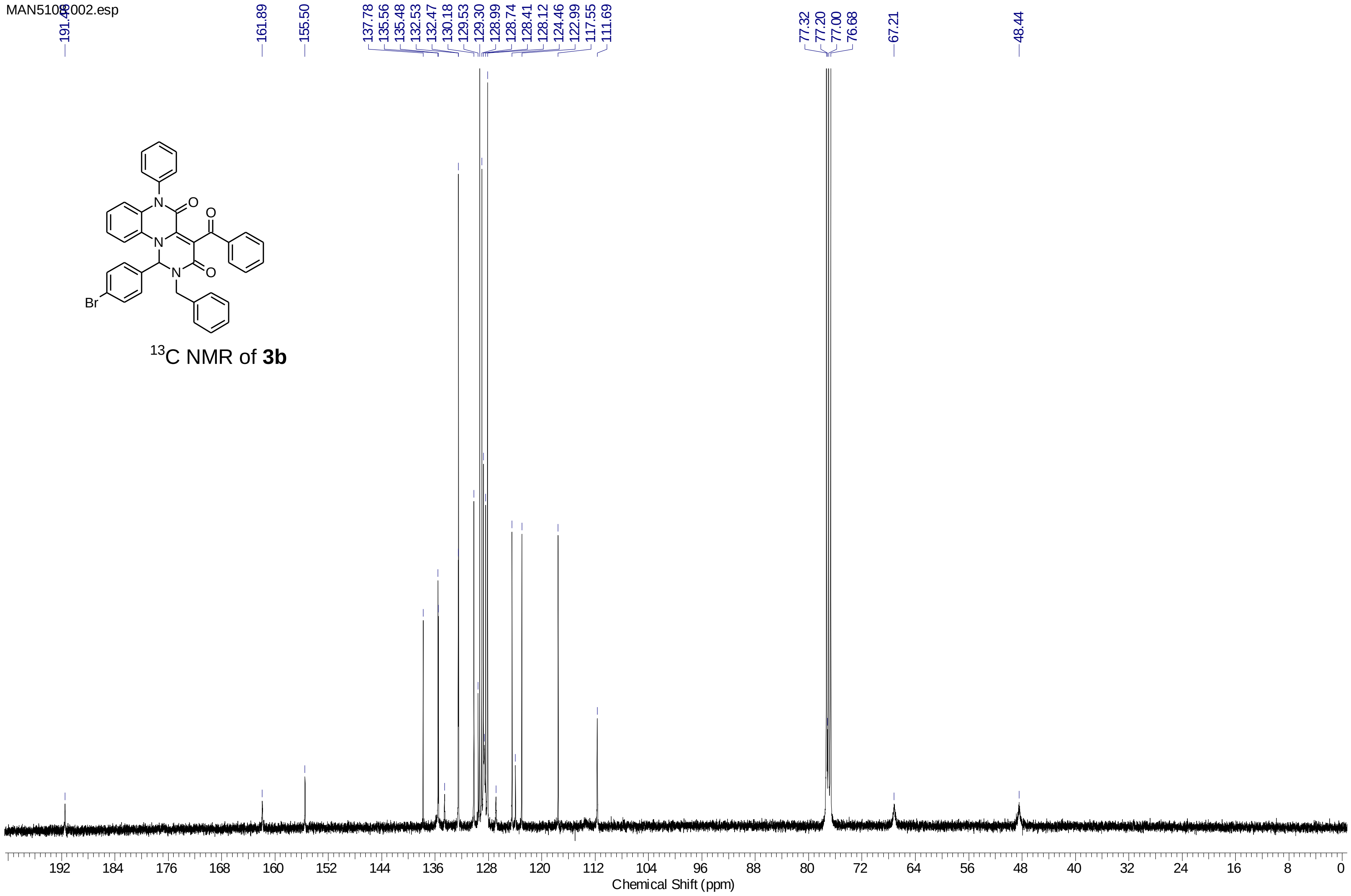
191.48
161.89
155.50
137.78
135.56
135.48
132.53
132.47
130.18
129.53
129.30
128.99
128.74
128.41
128.12
124.46
122.99
117.55
111.69
77.32
77.20
77.00
76.68
67.21
48.44
Br
N
O
Chemical Shift (ppm)

^{13}C NMR of **3b**

MAN5120.001.esp

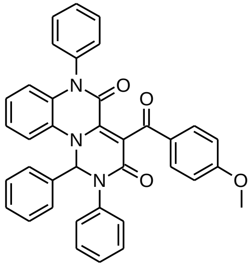

^{1}H NMR of **3c**

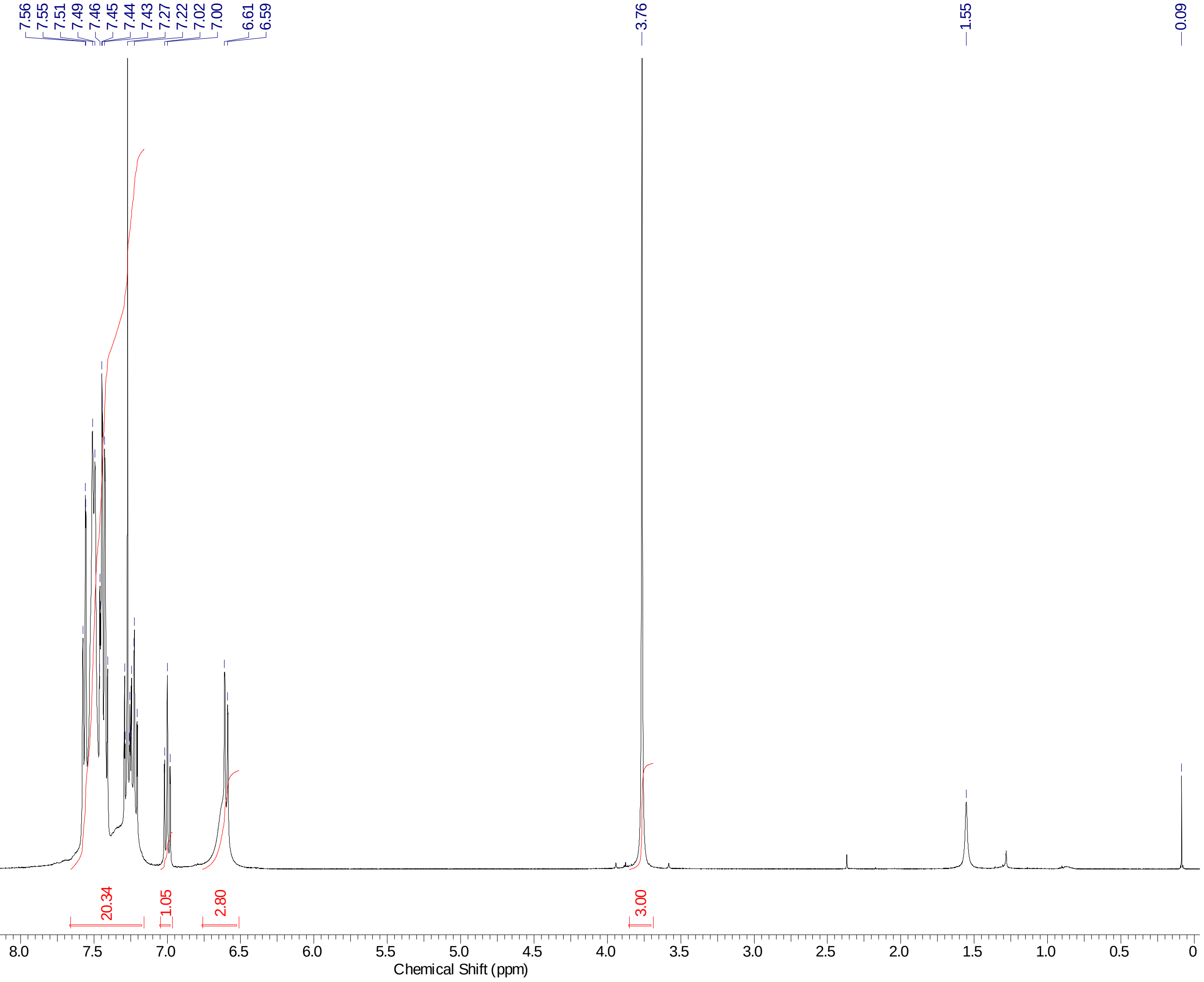

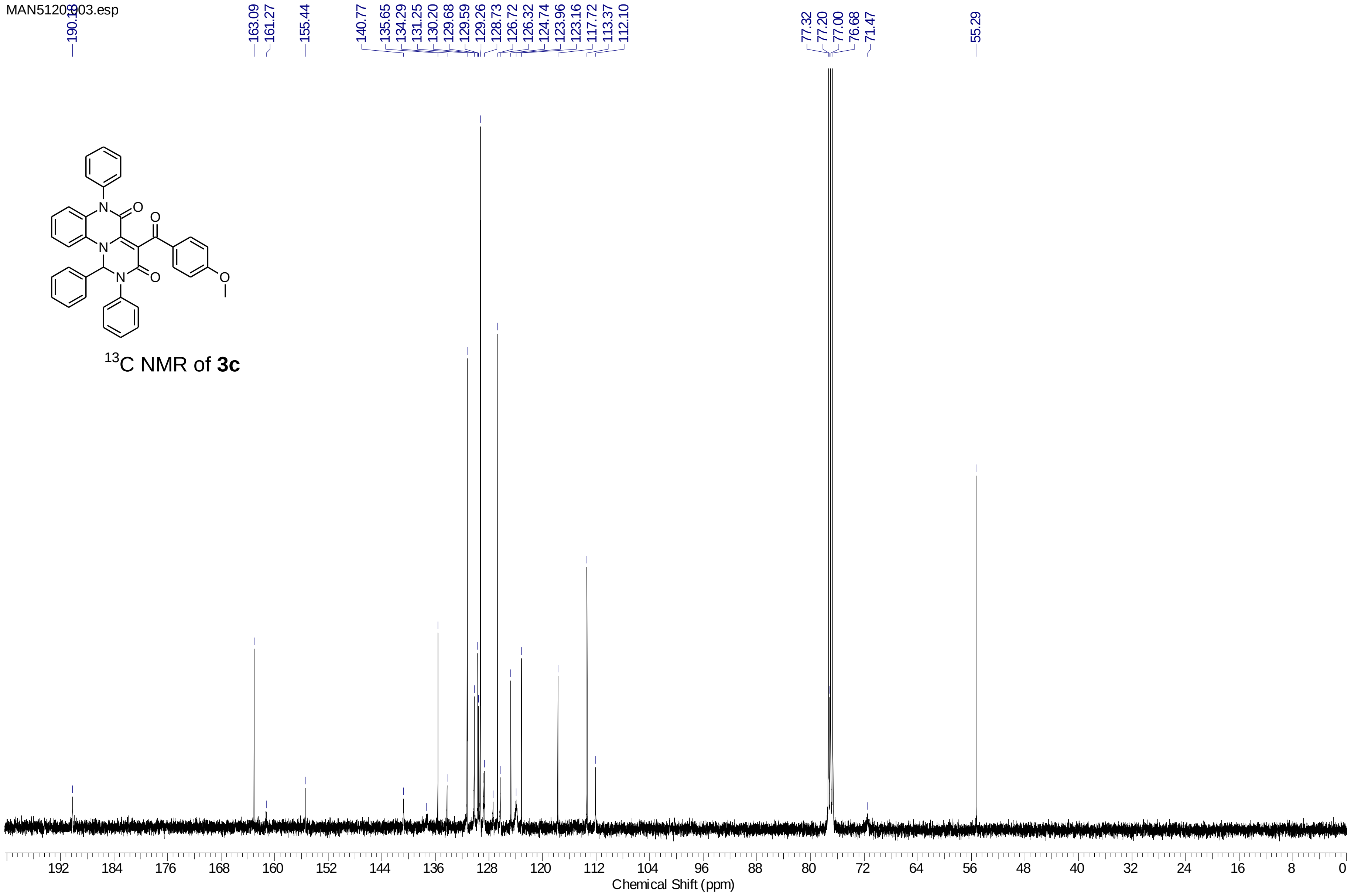
MAN5120903.esp
190.1
163.09
161.27
155.44
140.77
135.65
134.29
131.25
130.20
129.68
129.59
129.26
128.73
126.72
126.32
124.74
123.96
123.16
117.72
113.37
112.10
77.32
77.20
77.00
76.68
71.47
55.29
13C NMR of 3c
192 184 176 168 160 152 144 136 128 120 112 104 96 88 80 72 64 56 48 40 32 24 16 8 0
Chemical Shift (ppm)

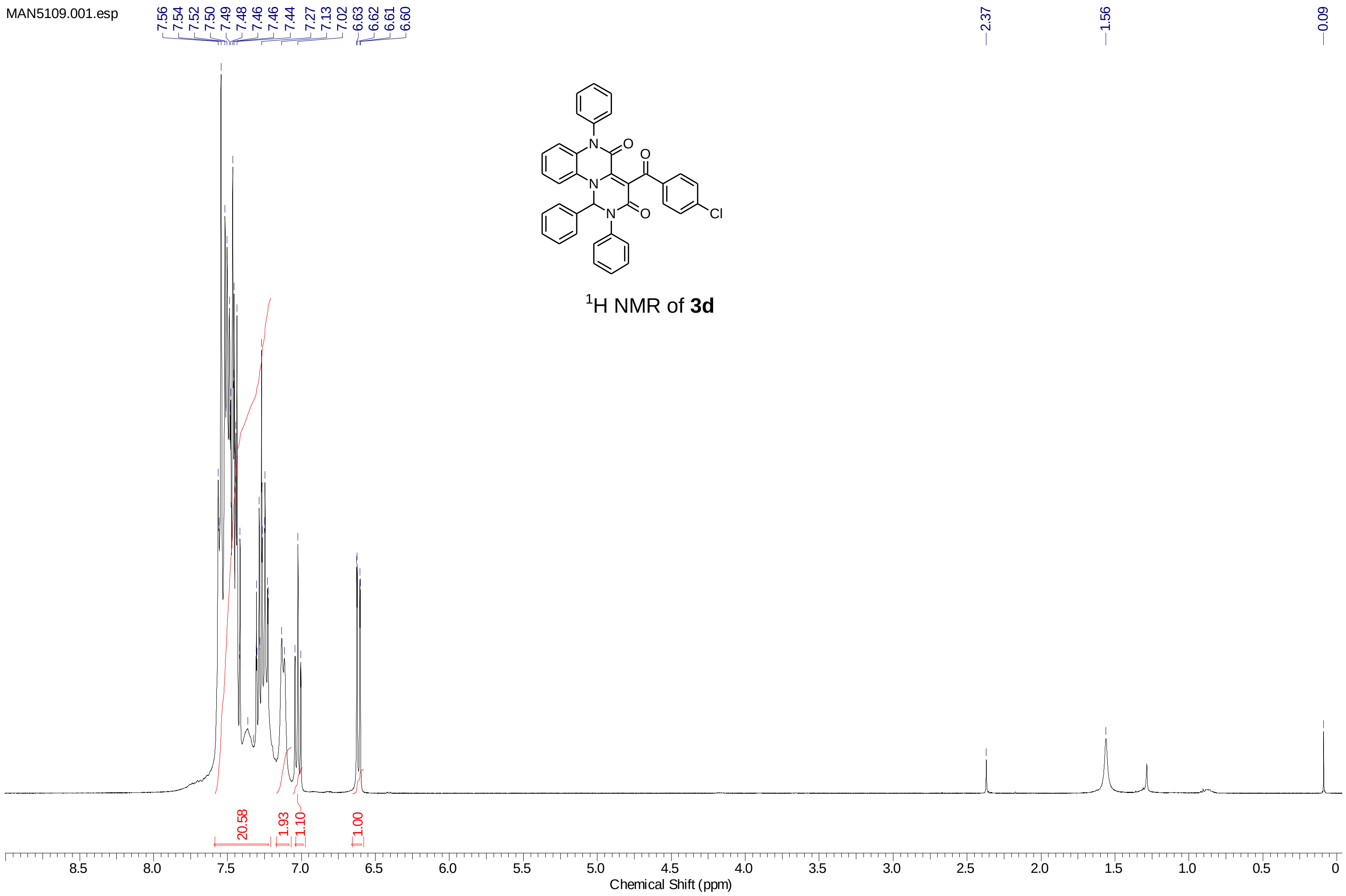
MAN5109.001.esp
7.56
7.54
7.52
7.50
7.49
7.48
7.46
7.46
7.44
7.27
7.13
7.02
6.63
6.62
6.61
6.60
2.37
1.56
0.09
N
O
O
N
N
O
Cl
^{1}H NMR of **3d**
20.58
1.93
1.10
1.00
8.5
8.0
7.5
7.0
6.5
6.0
5.5
5.0
4.5
4.0
3.5
3.0
2.5
2.0
1.5
1.0
0.5
0
Chemical Shift (ppm)

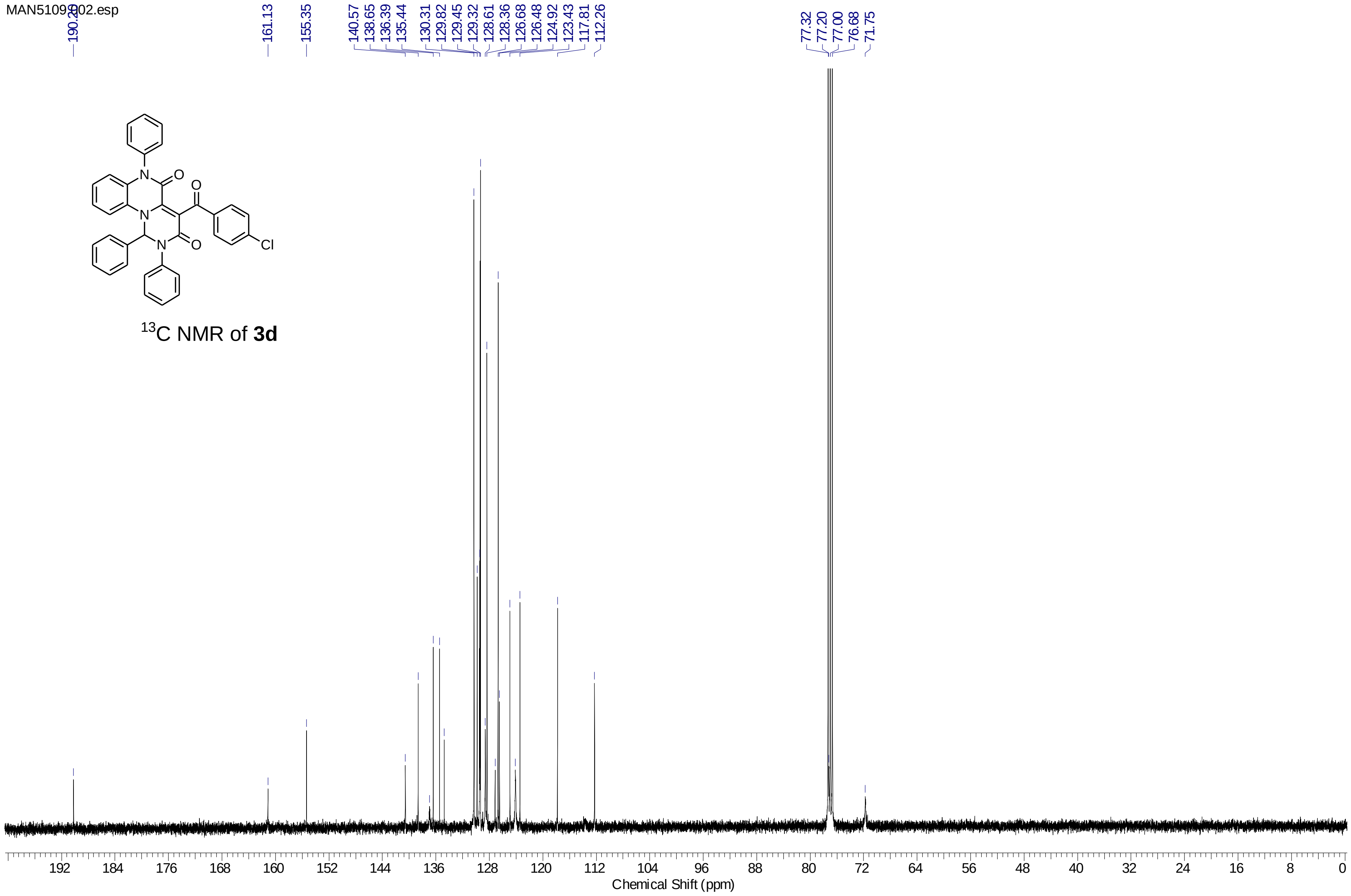
190.20
161.13
155.35
140.57
138.65
136.39
135.44
130.31
129.82
129.45
129.32
128.61
128.36
126.68
126.48
124.92
123.43
117.81
112.26
77.32
77.20
77.00
76.68
71.75
O
N
O
N
N
O
Cl
192 184 176 168 160 152 144 136 128 120 112 104 96 88 80 72 64 56 48 40 32 24 16 8 0
Chemical Shift (ppm)

^{13}C NMR of **3d**

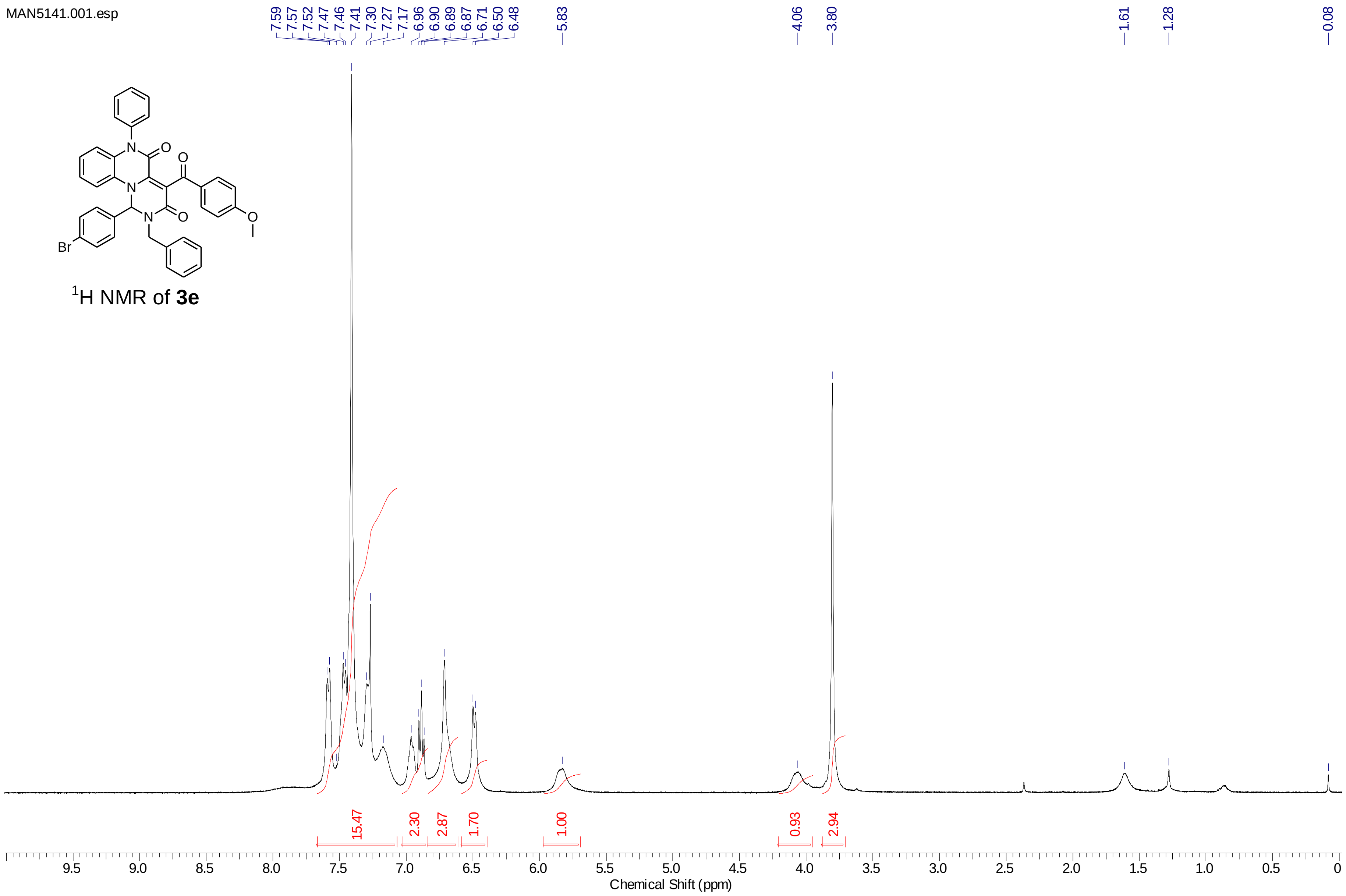
MAN5141.001.esp
7.59
7.57
7.52
7.47
7.46
7.41
7.30
7.27
7.17
6.96
6.90
6.89
6.87
6.71
6.50
6.48
5.83
4.06
3.80
1.61
1.28
0.08
Br
N
O
¹H NMR of 3e
15.47
2.30
2.87
1.70
1.00
0.93
2.94
Chemical Shift (ppm)

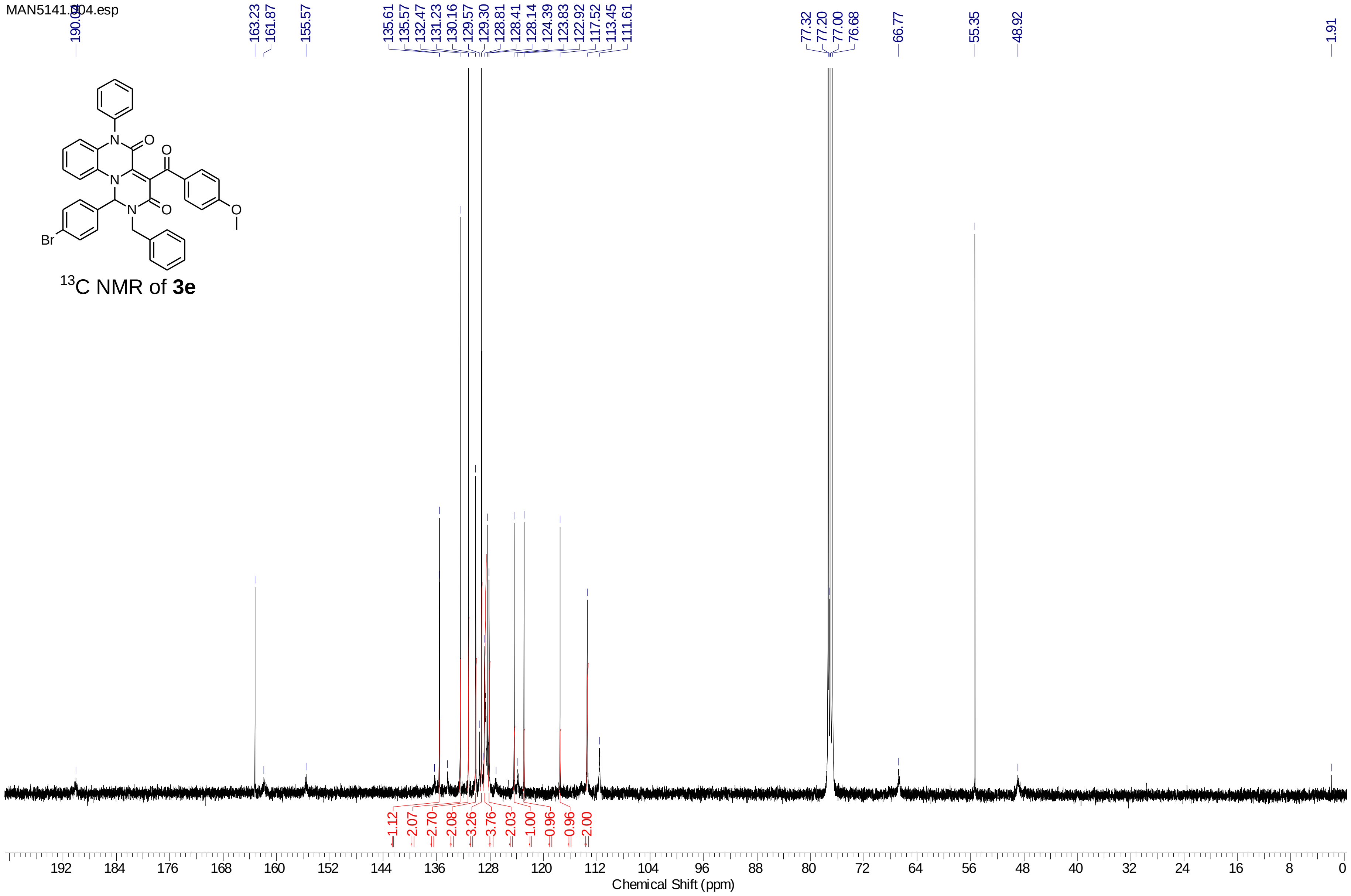
MAN5141.004.esp
190.04
163.23
161.87
155.57
135.61
135.57
132.47
131.23
130.16
129.57
129.30
128.81
128.41
128.14
124.39
123.83
122.92
117.52
113.45
111.61
77.32
77.20
77.00
76.68
66.77
55.35
48.92
1.91
^{13}C NMR of **3e**
1.12
2.07
2.70
2.08
3.26
3.76
2.03
1.00
0.96
0.96
2.00
192 184 176 168 160 152 144 136 128 120 112 104 96 88 80 72 64 56 48 40 32 24 16 8 0
Chemical Shift (ppm)

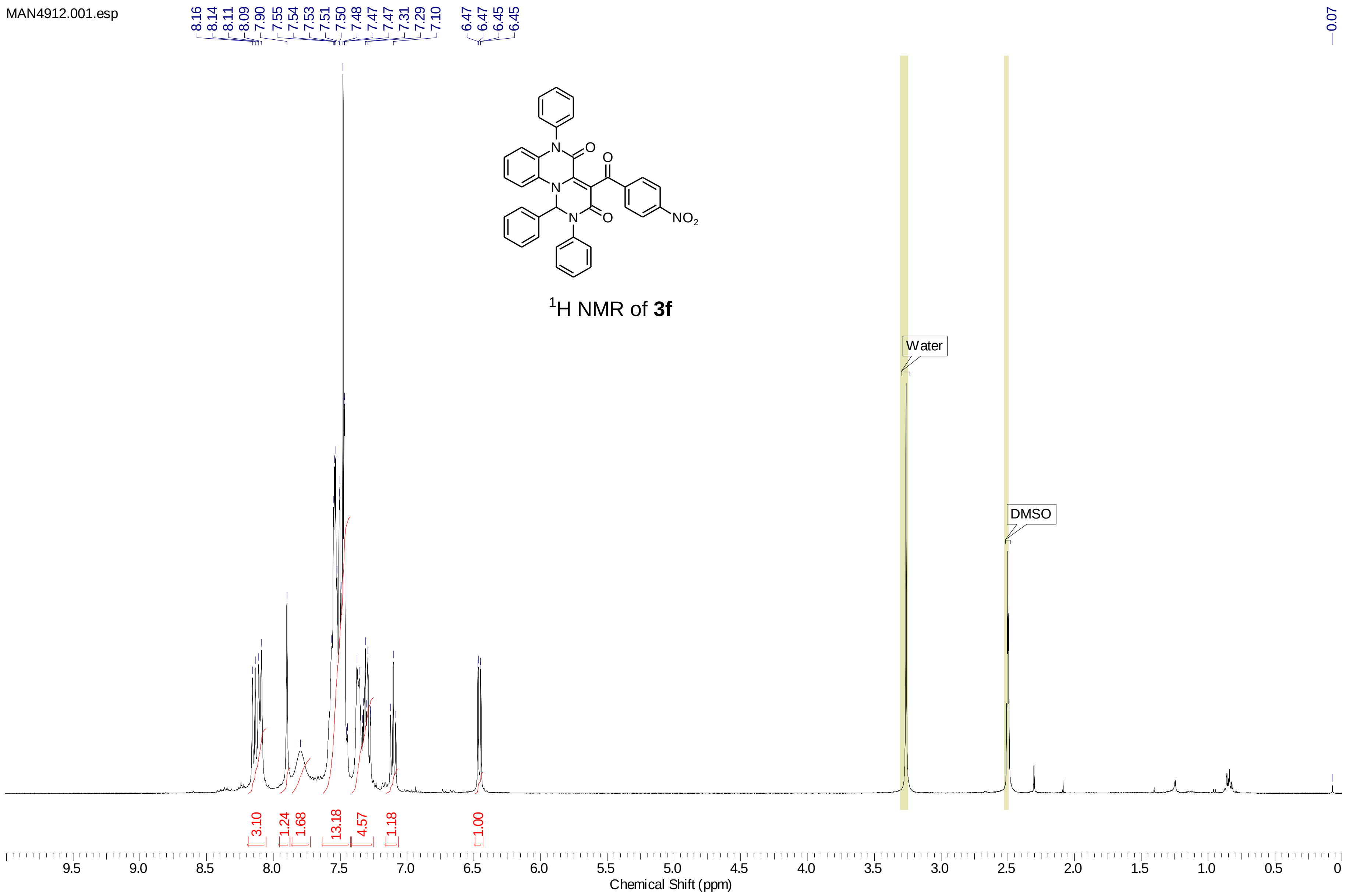
MAN4912.001.esp
8.16
8.14
8.11
8.09
7.90
7.55
7.54
7.53
7.51
7.50
7.48
7.47
7.47
7.31
7.29
7.10
6.47
6.47
6.45
6.45
0.07
NO2
¹H NMR of 3f
Water
DMSO
3.10
1.24
1.68
13.18
4.57
1.18
1.00
9.5 9.0 8.5 8.0 7.5 7.0 6.5 6.0 5.5 5.0 4.5 4.0 3.5 3.0 2.5 2.0 1.5 1.0 0.5 0
Chemical Shift (ppm)

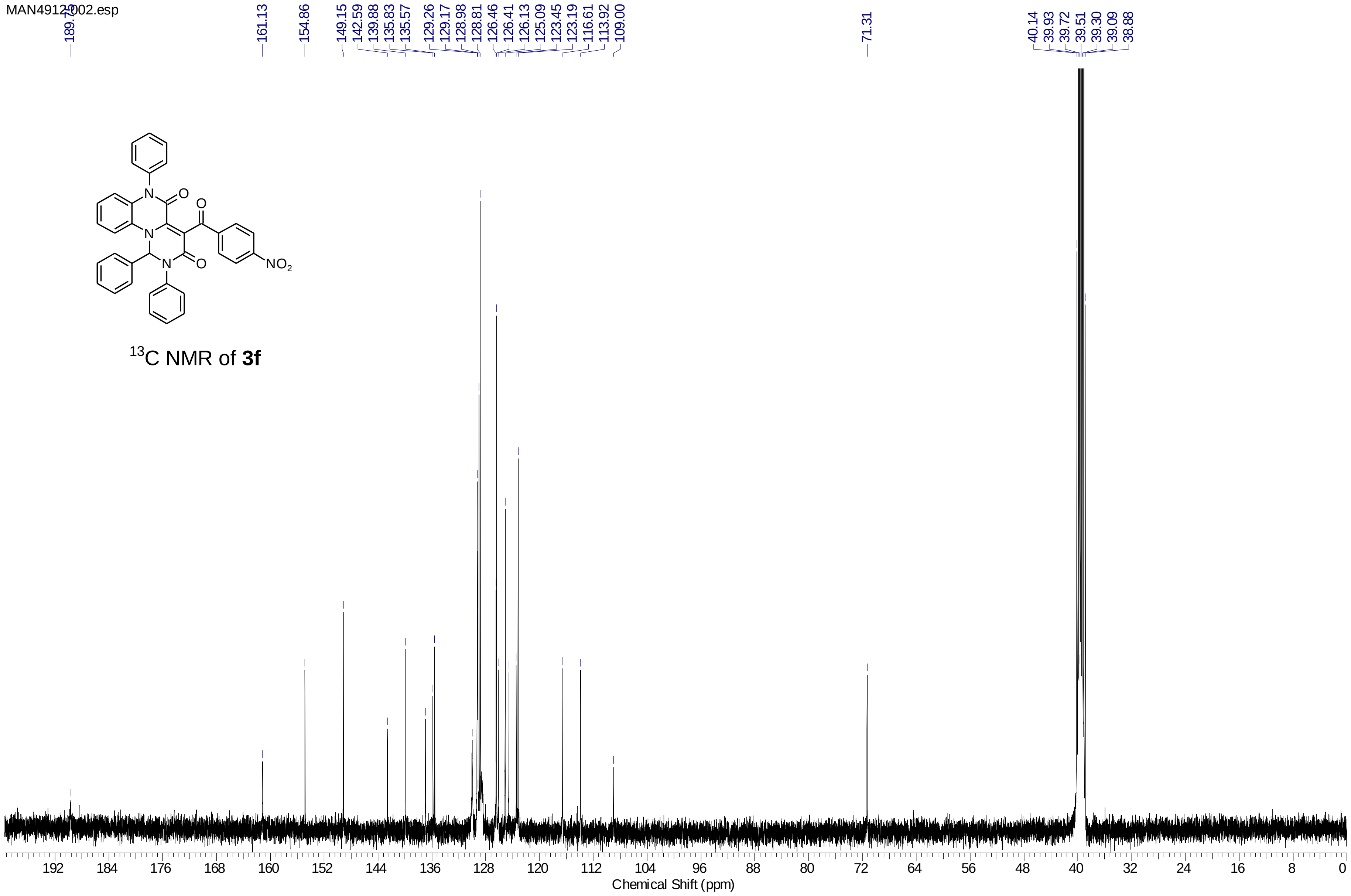
MAN4912.002.esp
189.75
161.13
154.86
149.15
142.59
139.88
135.83
135.57
129.26
129.17
128.98
128.81
126.46
126.41
126.13
125.09
123.45
123.19
116.61
113.92
109.00
71.31
40.14
39.93
39.72
39.51
39.30
39.09
38.88
N
O
O
N
N
O
NO_2
192 184 176 168 160 152 144 136 128 120 112 104 96 88 80 72 64 56 48 40 32 24 16 8 0
Chemical Shift (ppm)

^{13}C NMR of **3f**

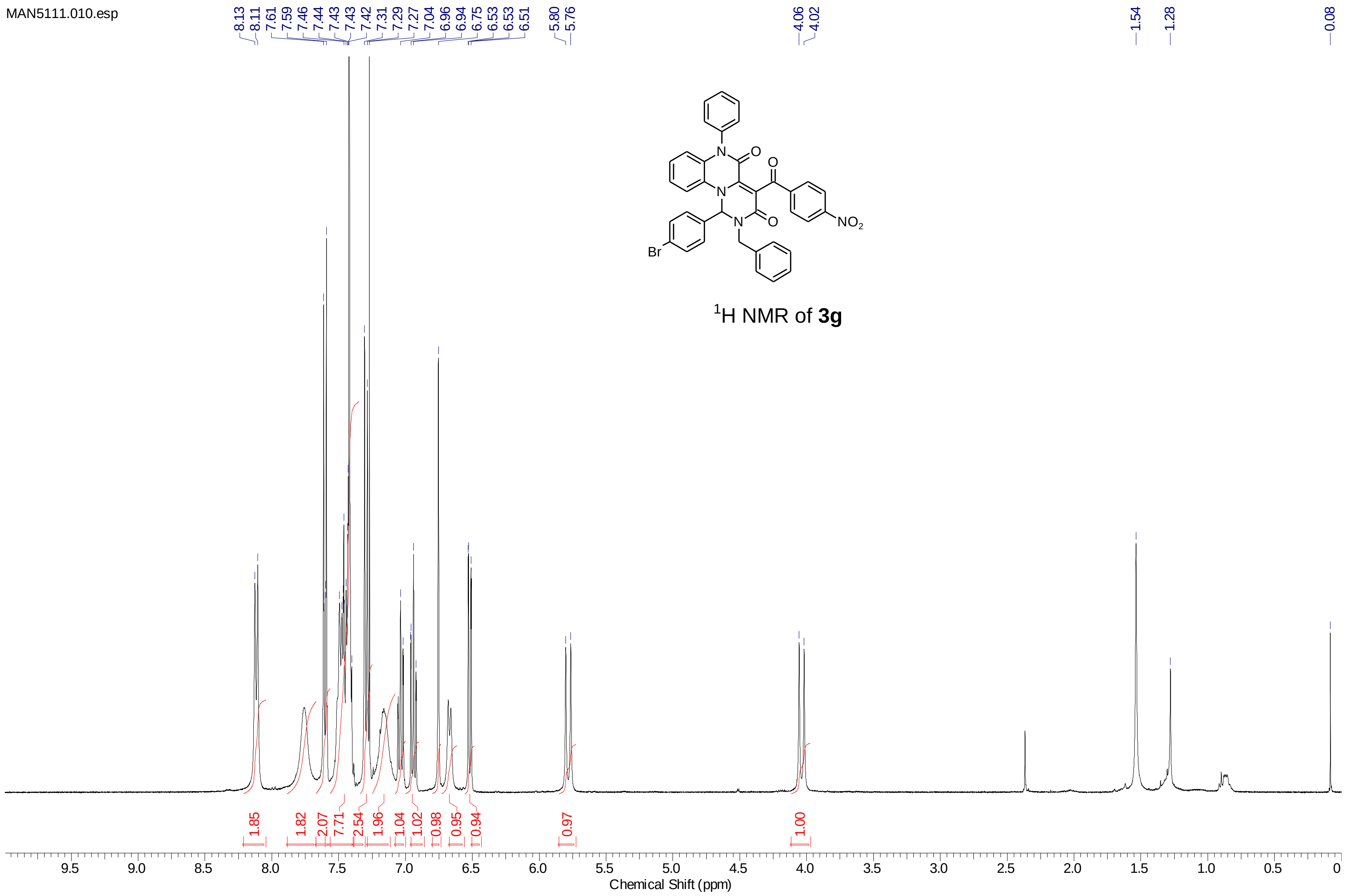
MAN5111.010.esp
8.13
8.11
7.61
7.59
7.46
7.44
7.43
7.43
7.42
7.31
7.29
7.27
7.04
6.96
6.94
6.75
6.53
6.53
6.51
5.80
5.76
4.06
4.02
1.54
1.28
0.08
N
O
O
O
N
N
Br
NO2
1H NMR of 3g
1.85
1.82
2.07
7.71
2.54
1.96
1.04
1.02
0.98
0.95
0.94
0.97
1.00
Chemical Shift (ppm)

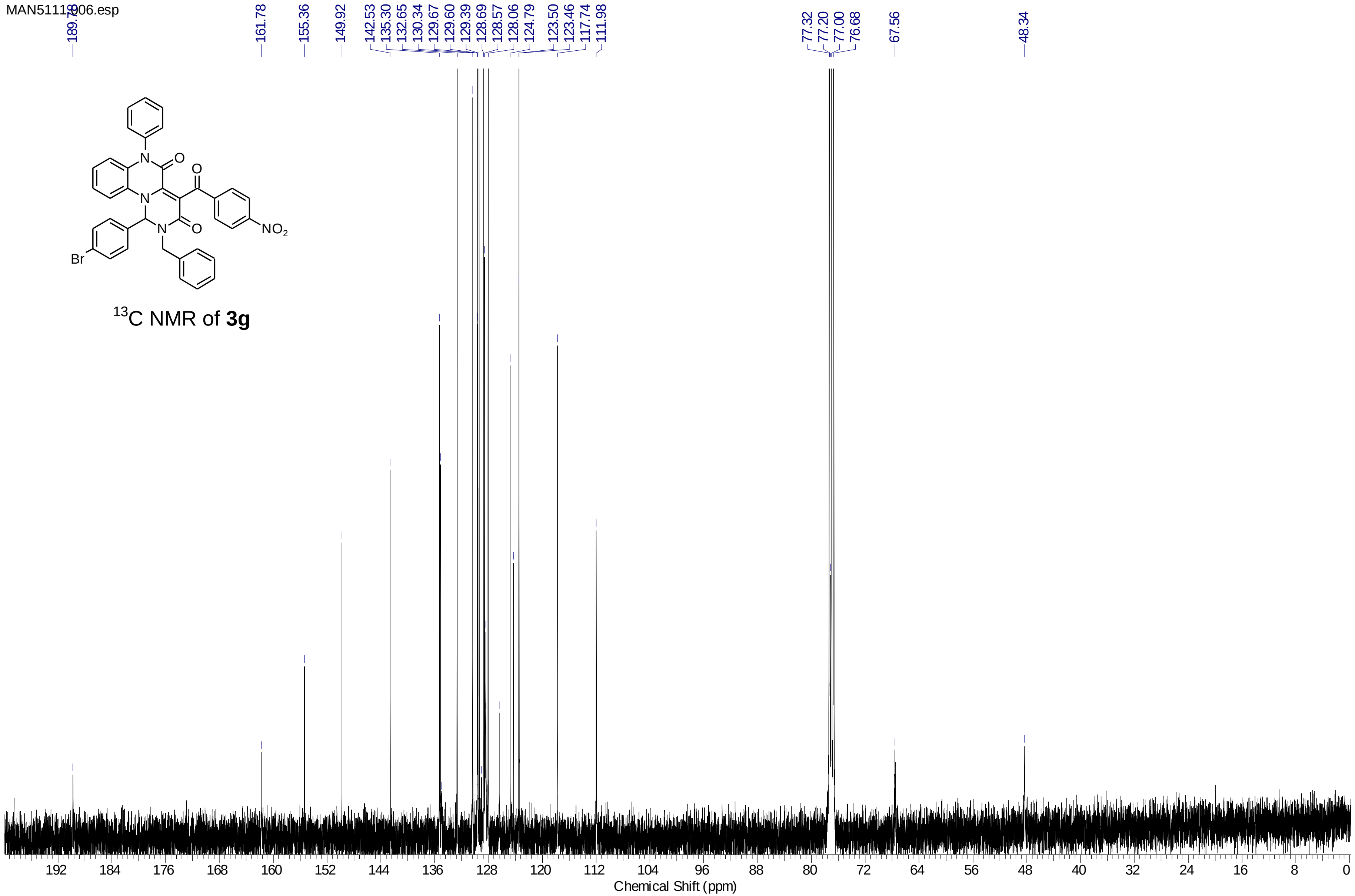
189.78
161.78
155.36
149.92
142.53
135.30
132.65
130.34
129.67
129.60
129.39
128.69
128.57
128.06
124.79
123.50
123.46
117.74
111.98
77.32
77.20
77.00
76.68
67.56
48.34
NO2
Br
192 184 176 168 160 152 144 136 128 120 112 104 96 88 80 72 64 56 48 40 32 24 16 8 0
Chemical Shift (ppm)

^{13}C NMR of **3g**

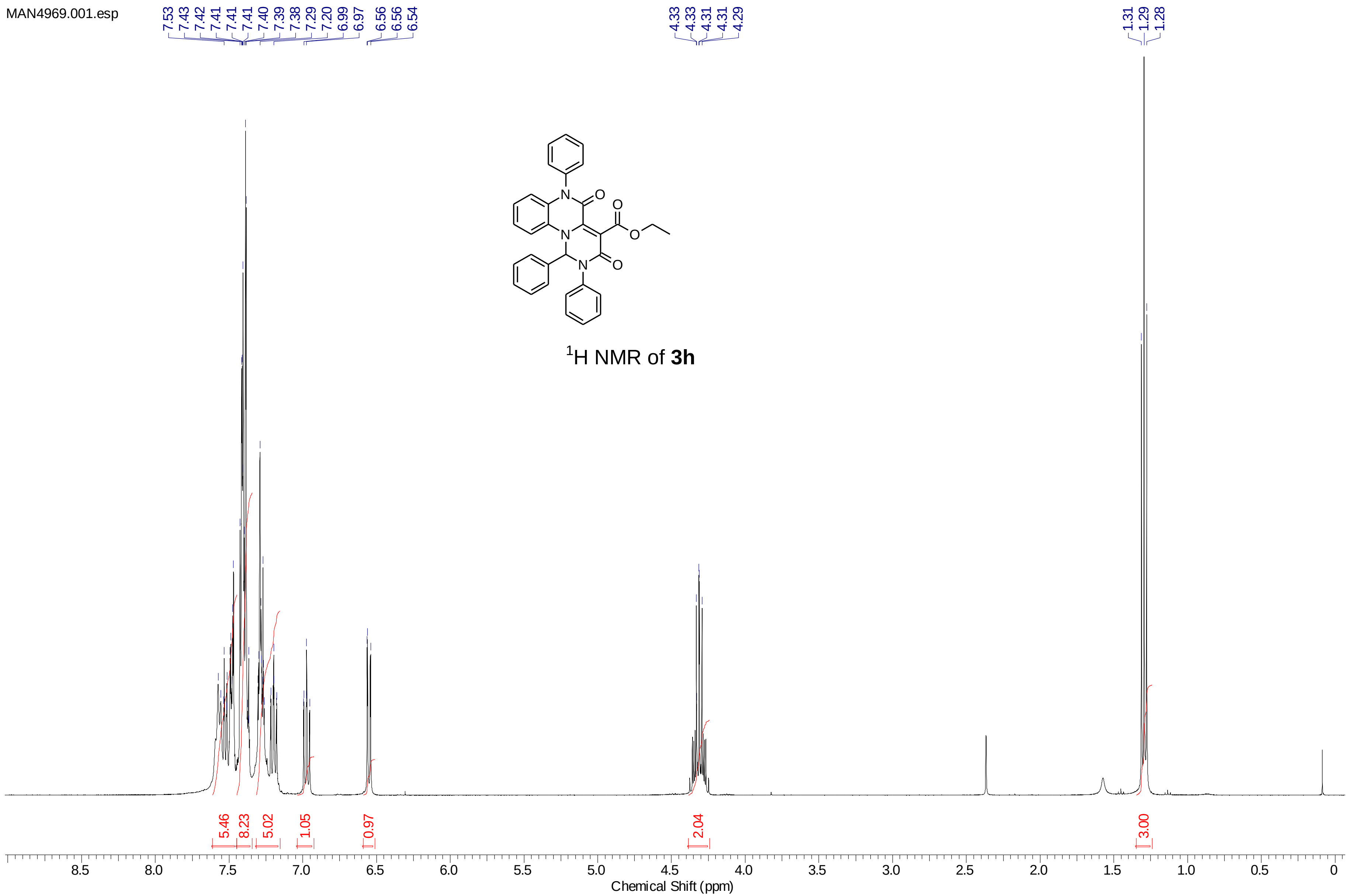
MAN4969.001.esp
7.53
7.43
7.42
7.41
7.41
7.41
7.40
7.39
7.38
7.29
7.20
6.99
6.97
6.56
6.56
6.54
4.33
4.33
4.31
4.31
4.29
1.31
1.29
1.28
N
O
O
O
N
N
O
^{1}H NMR of **3h**
5.46
8.23
5.02
1.05
0.97
2.04
3.00
8.5
8.0
7.5
7.0
6.5
6.0
5.5
5.0
4.5
4.0
3.5
3.0
2.5
2.0
1.5
1.0
0.5
0
Chemical Shift (ppm)

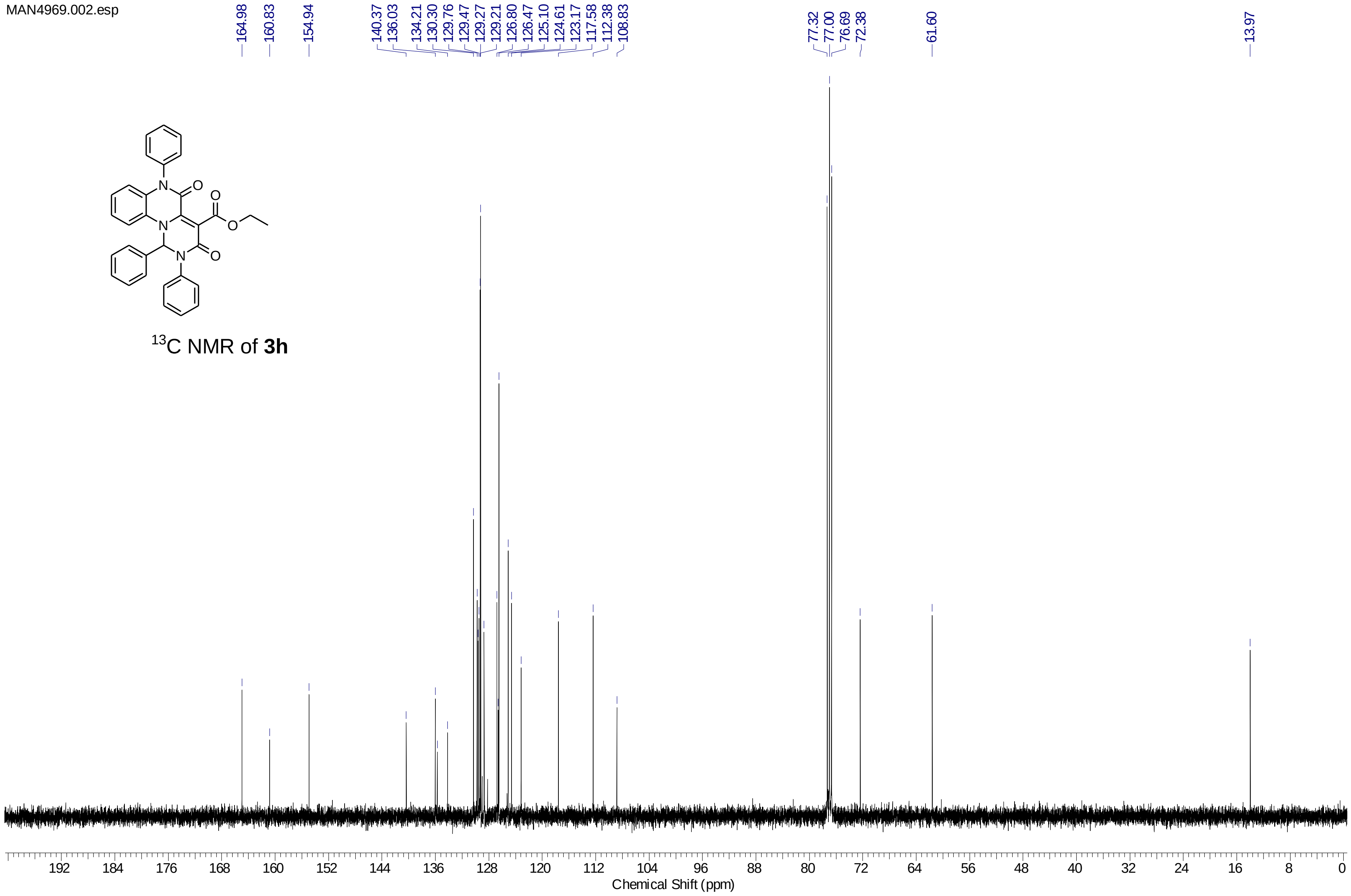
MAN4969.002.esp
164.98
160.83
154.94
140.37
136.03
134.21
130.30
129.76
129.47
129.27
129.21
126.80
126.47
125.10
124.61
123.17
117.58
112.38
108.83
77.32
77.00
76.69
72.38
61.60
13.97
192 184 176 168 160 152 144 136 128 120 112 104 96 88 80 72 64 56 48 40 32 24 16 8 0
Chemical Shift (ppm)

^{13}C NMR of **3h**

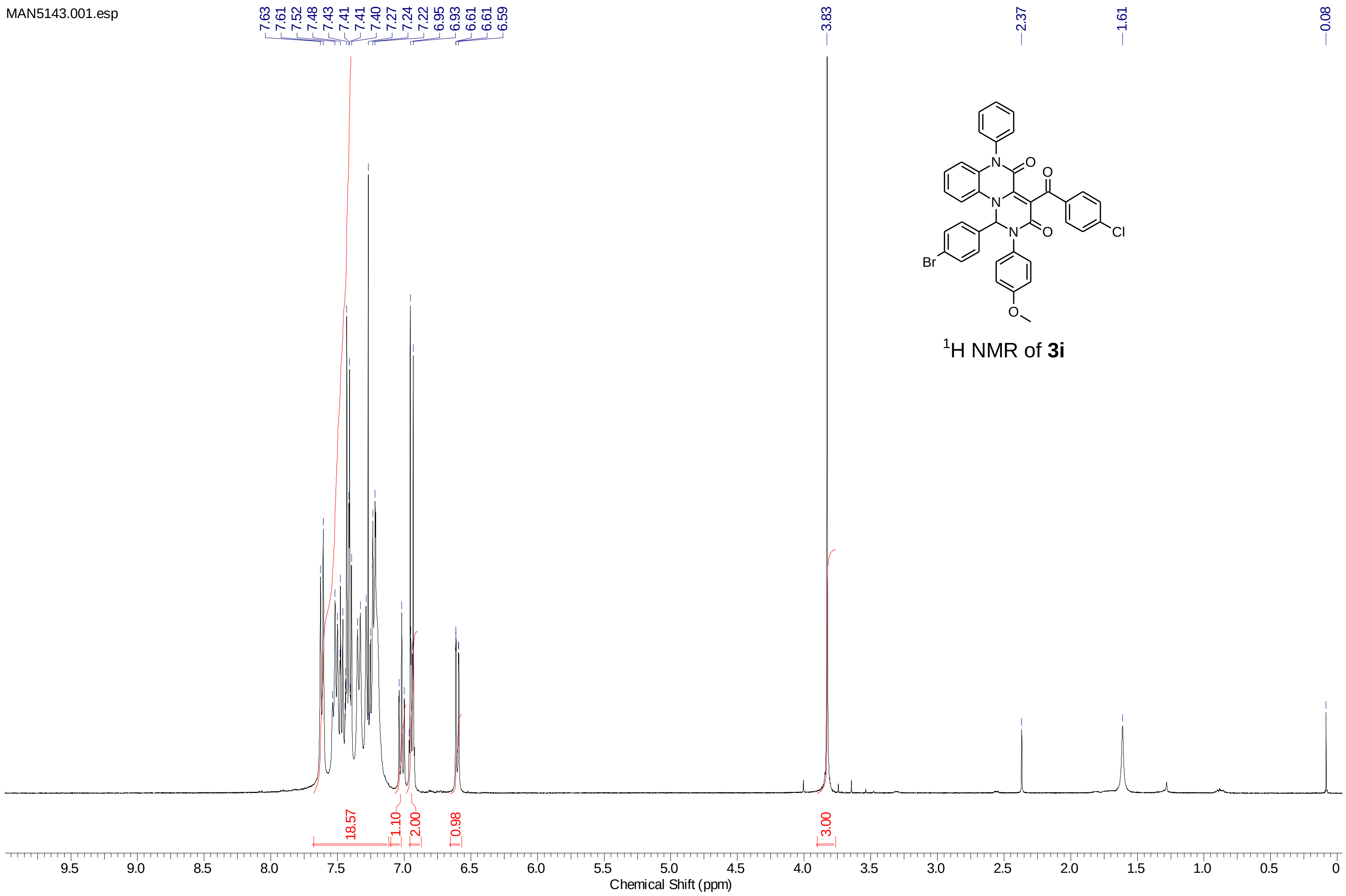
MAN5143.001.esp
7.63
7.61
7.52
7.48
7.43
7.41
7.41
7.40
7.27
7.24
7.22
6.95
6.93
6.61
6.61
6.59
3.83
2.37
1.61
0.08
18.57
1.10
2.00
0.98
3.00
9.5
9.0
8.5
8.0
7.5
7.0
6.5
6.0
5.5
5.0
4.5
4.0
3.5
3.0
2.5
2.0
1.5
1.0
0.5
0
Chemical Shift (ppm)
N
O
O
N
N
O
Cl
Br
O

^{1}H NMR of **3i**

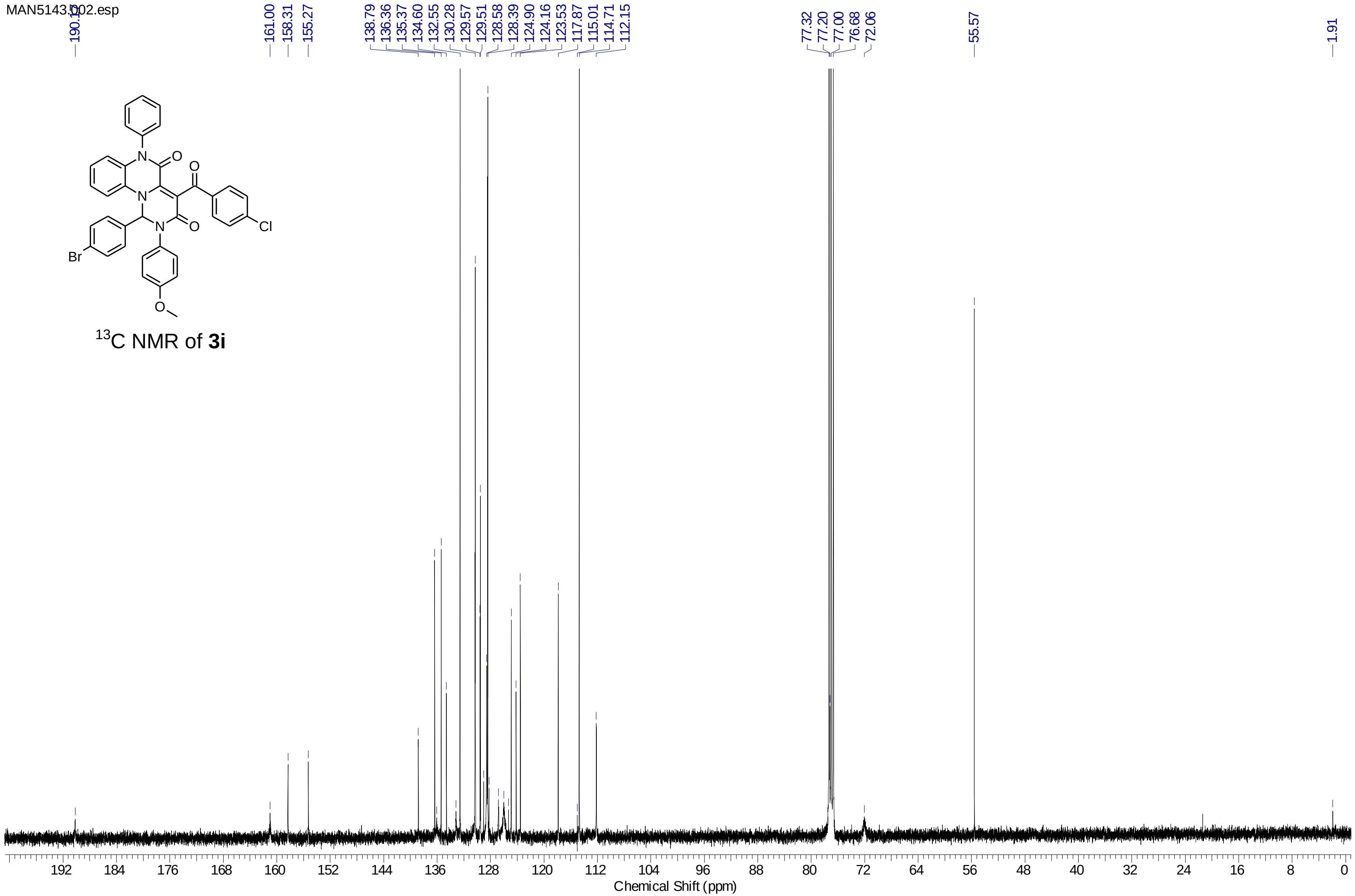
MAN5143.002.esp
190.17
161.00
158.31
155.27
138.79
136.36
135.37
134.60
132.55
130.28
129.57
129.51
128.58
128.39
124.90
124.16
123.53
117.87
115.01
114.71
112.15
77.32
77.20
77.00
76.68
72.06
55.57
1.91
O
N
O
N
N
O
Cl
Br
O
192
184
176
168
160
152
144
136
128
120
112
104
96
88
80
72
64
56
48
40
32
24
16
8
0
Chemical Shift (ppm)

^{13}C NMR of **3i**

MAN5113.001.esp

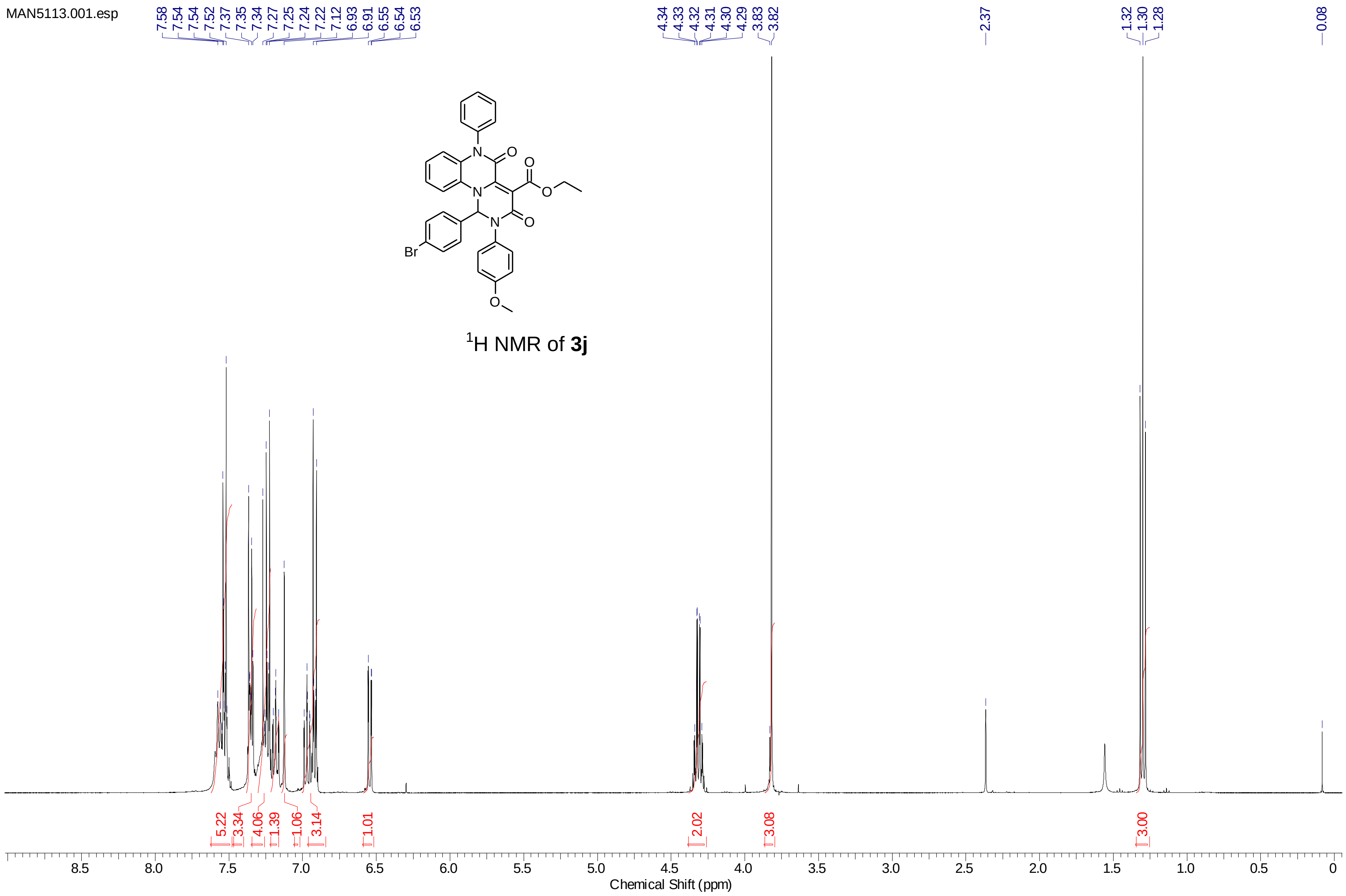

^{1}H NMR of **3j**

MAN5113.002.esp

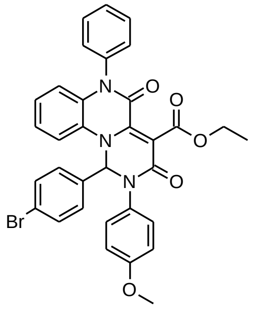

^{13}C NMR of **3j**

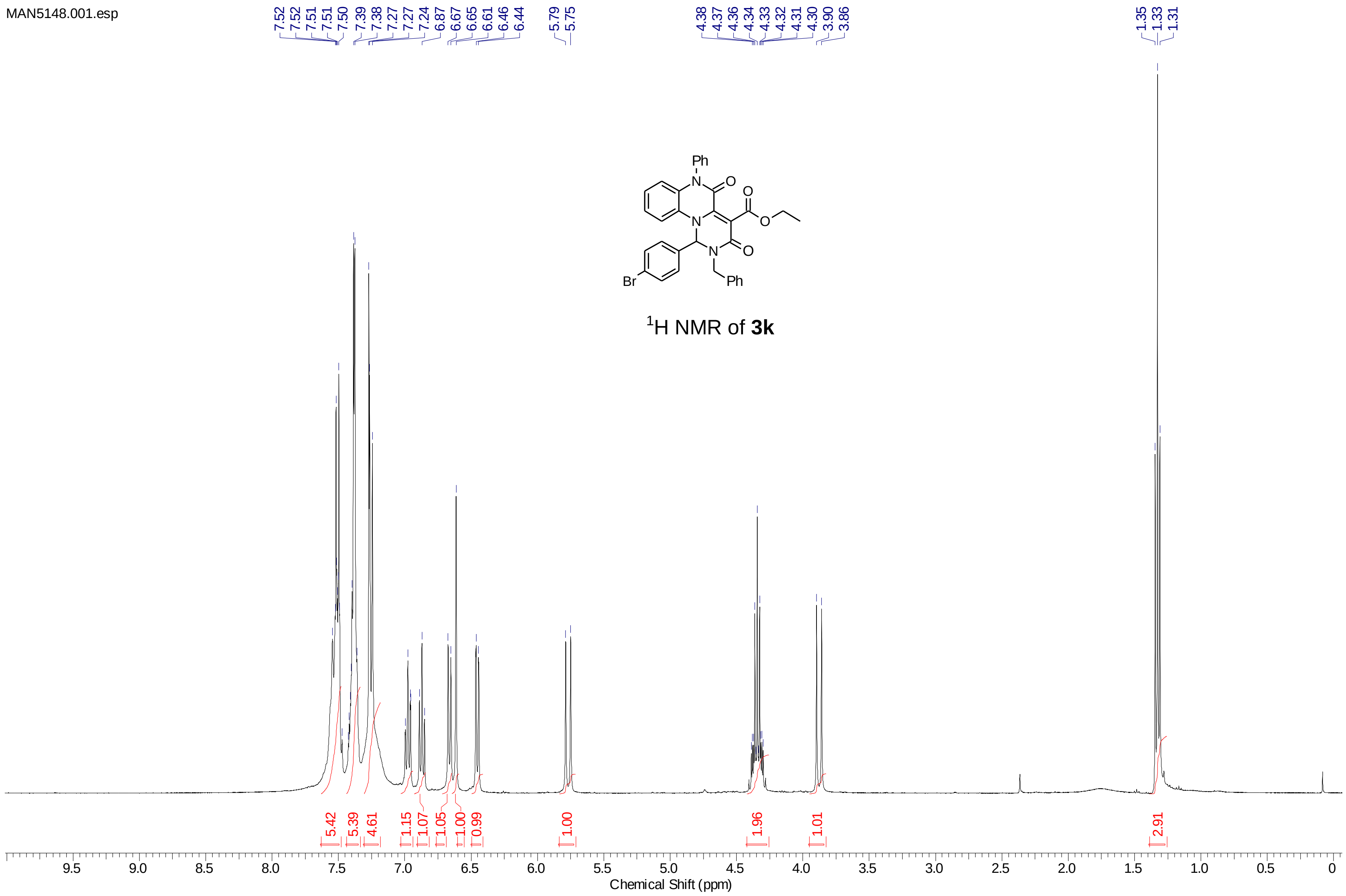
MAN5148.001.esp
7.52
7.52
7.51
7.51
7.50
7.39
7.38
7.27
7.27
7.24
6.87
6.67
6.65
6.61
6.46
6.44
5.79
5.75
4.38
4.37
4.36
4.34
4.33
4.32
4.31
4.30
3.90
3.86
1.35
1.33
1.31
Ph
N
O
O
O
N
N
O
Br
Ph
¹H NMR of **3k**
5.42
5.39
4.61
1.15
1.07
1.05
1.00
0.99
1.00
1.96
1.01
2.91
9.5
9.0
8.5
8.0
7.5
7.0
6.5
6.0
5.5
5.0
4.5
4.0
3.5
3.0
2.5
2.0
1.5
1.0
0.5
0
Chemical Shift (ppm)

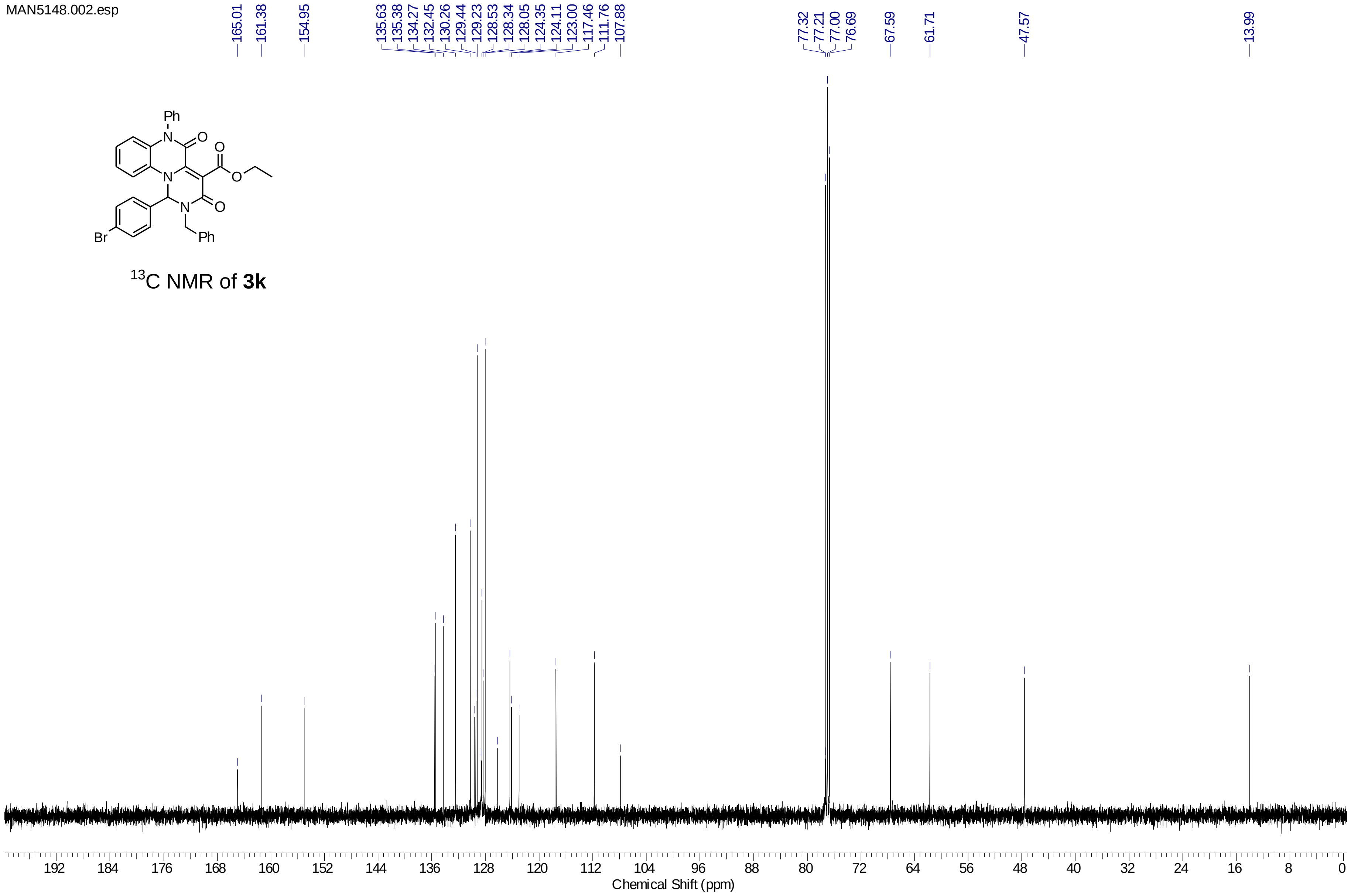
MAN5148.002.esp
165.01
161.38
154.95
135.63
135.38
134.27
132.45
130.26
129.44
129.23
128.53
128.34
128.05
124.35
124.11
123.00
117.46
111.76
107.88
77.32
77.21
77.00
76.69
67.59
61.71
47.57
13.99
Ph
N
O
O
N
O
N
O
Br
Ph
192 184 176 168 160 152 144 136 128 120 112 104 96 88 80 72 64 56 48 40 32 24 16 8 0
Chemical Shift (ppm)

^{13}C NMR of **3k**

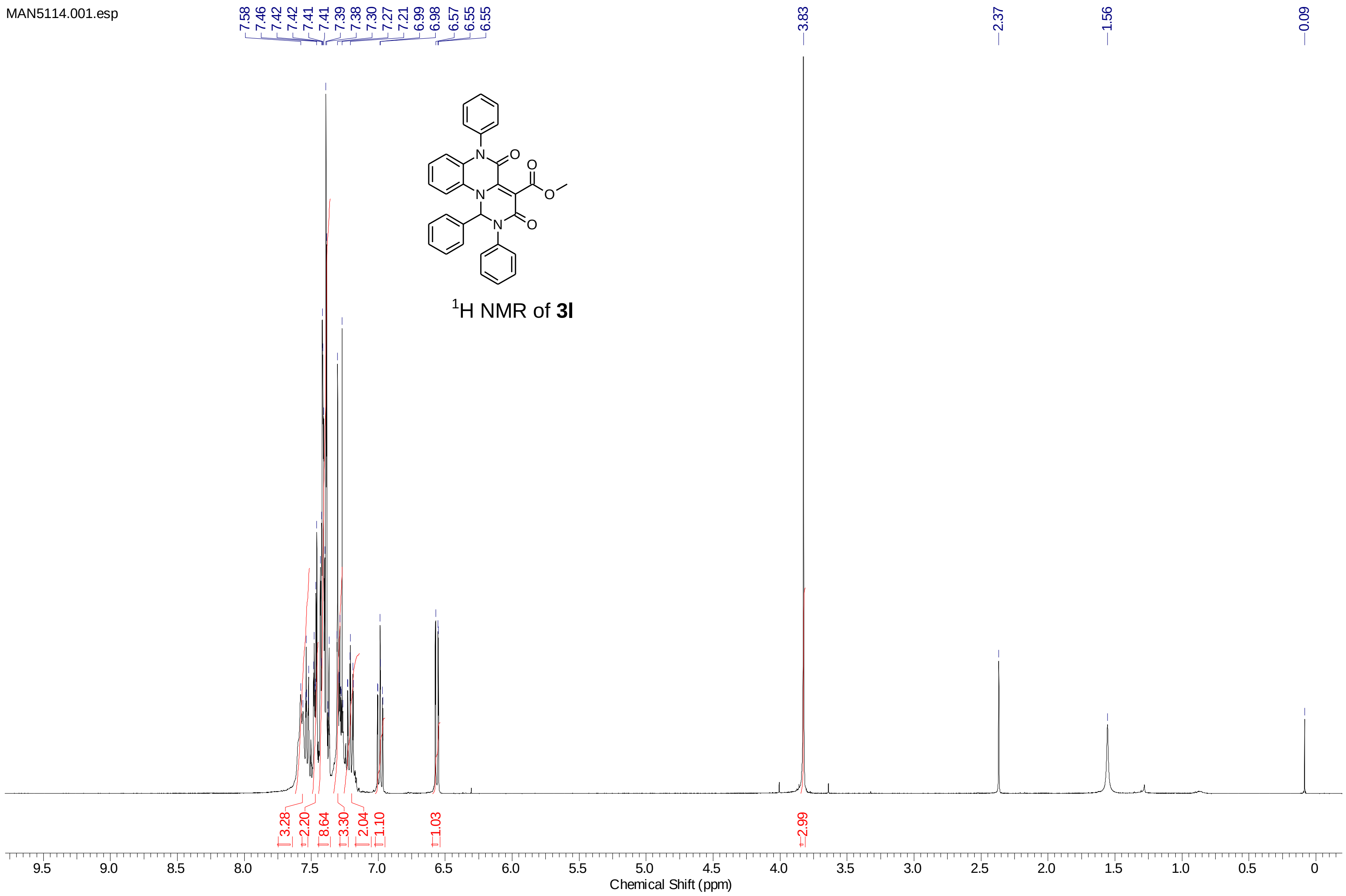
MAN5114.001.esp
7.58
7.46
7.42
7.42
7.41
7.41
7.39
7.38
7.30
7.27
7.21
6.99
6.98
6.57
6.55
6.55
3.83
2.37
1.56
0.09
^{1}H NMR of 3l
3.28
2.20
8.64
3.30
2.04
1.10
1.03
2.99
9.5
9.0
8.5
8.0
7.5
7.0
6.5
6.0
5.5
5.0
4.5
4.0
3.5
3.0
2.5
2.0
1.5
1.0
0.5
0
Chemical Shift (ppm)

MAN5114.002.esp

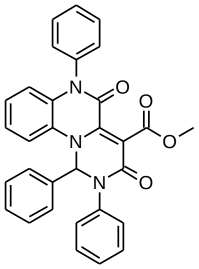

^{13}C NMR of **3l**

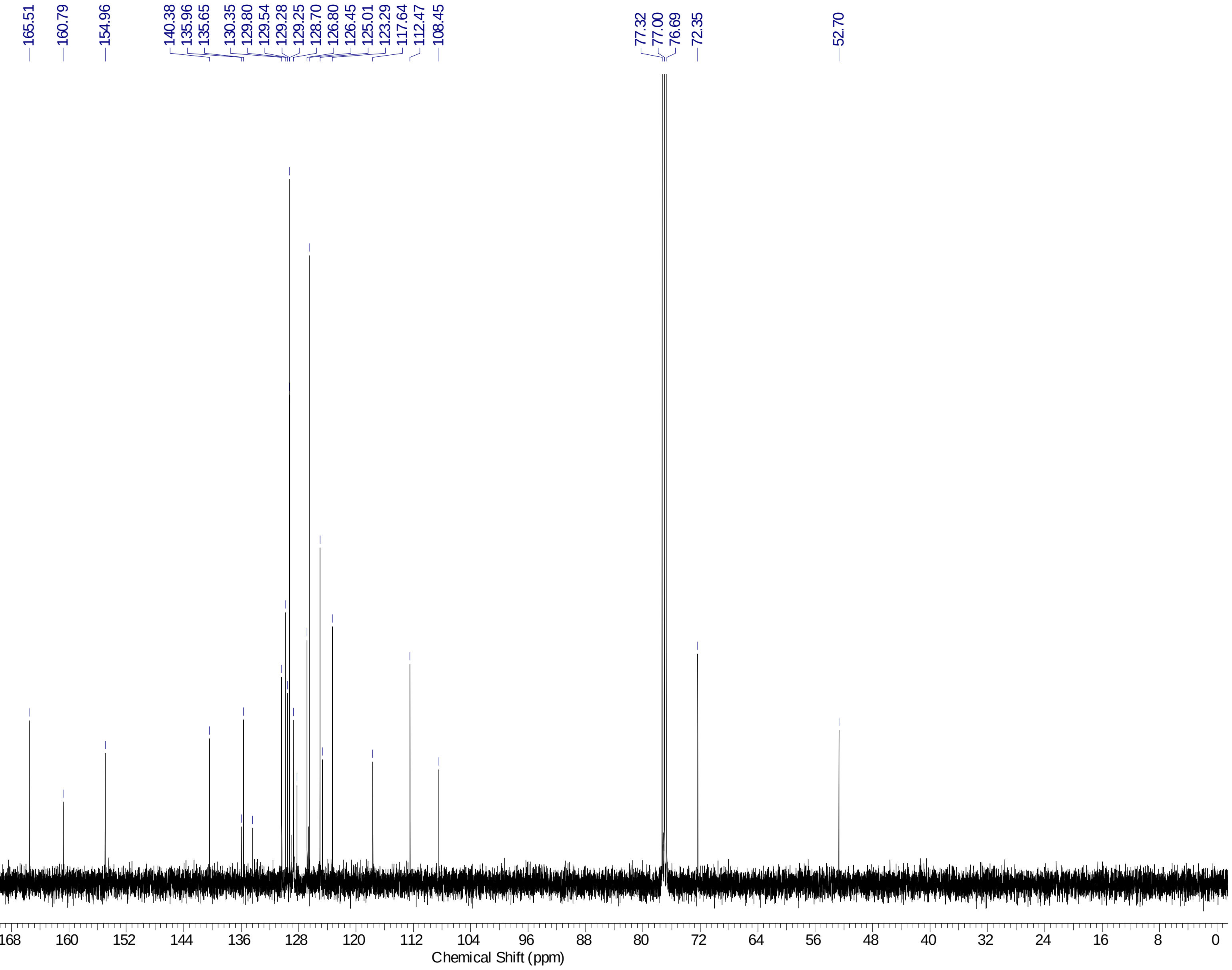

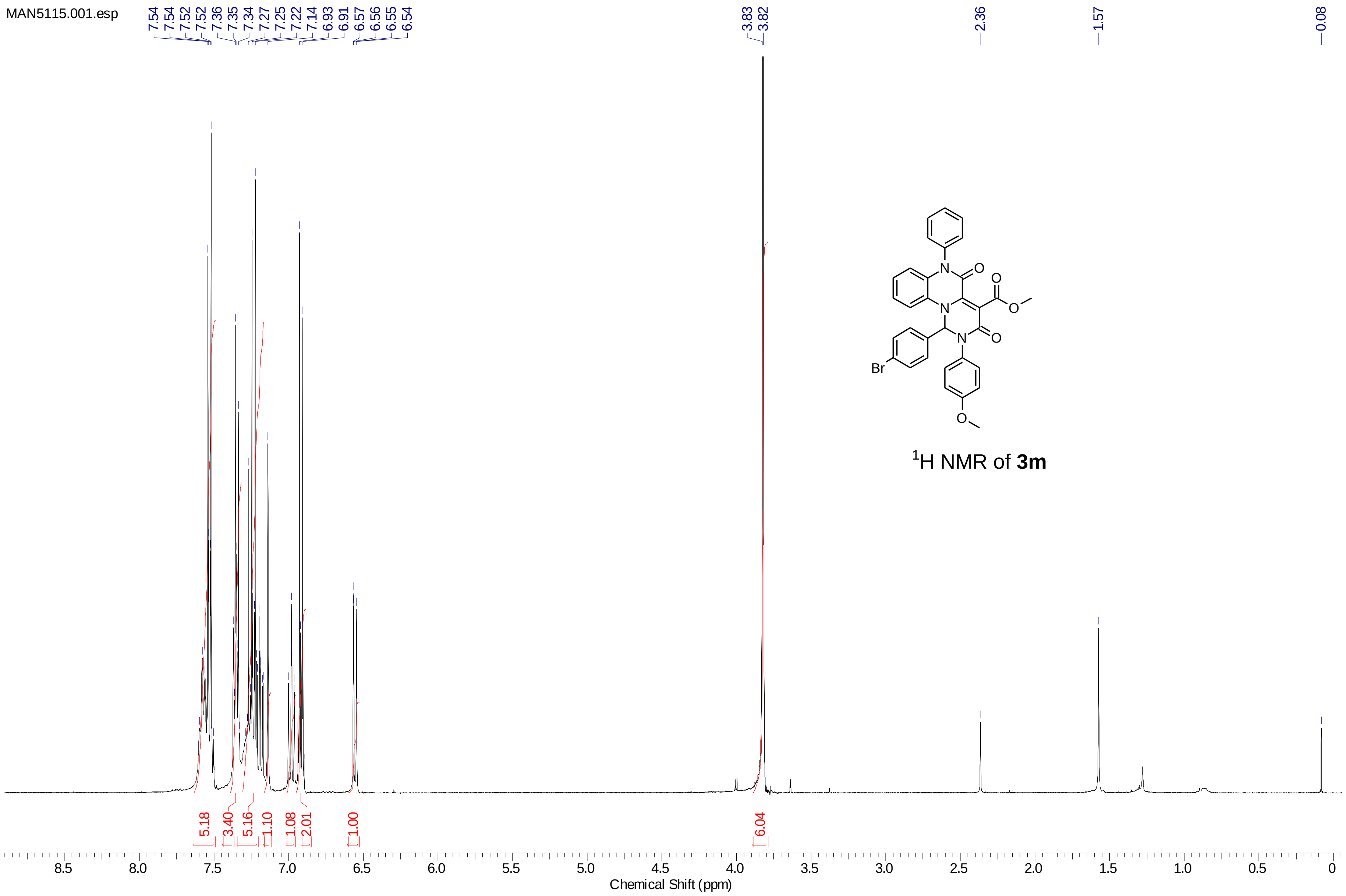
MAN5115.001.esp
7.54
7.54
7.52
7.52
7.36
7.35
7.34
7.27
7.25
7.22
7.14
6.93
6.91
6.57
6.56
6.55
6.54
3.83
3.82
2.36
1.57
0.08
Br
N
O
5.18
3.40
5.16
1.10
1.08
2.01
1.00
6.04
Chemical Shift (ppm)

^{1}H NMR of **3m**

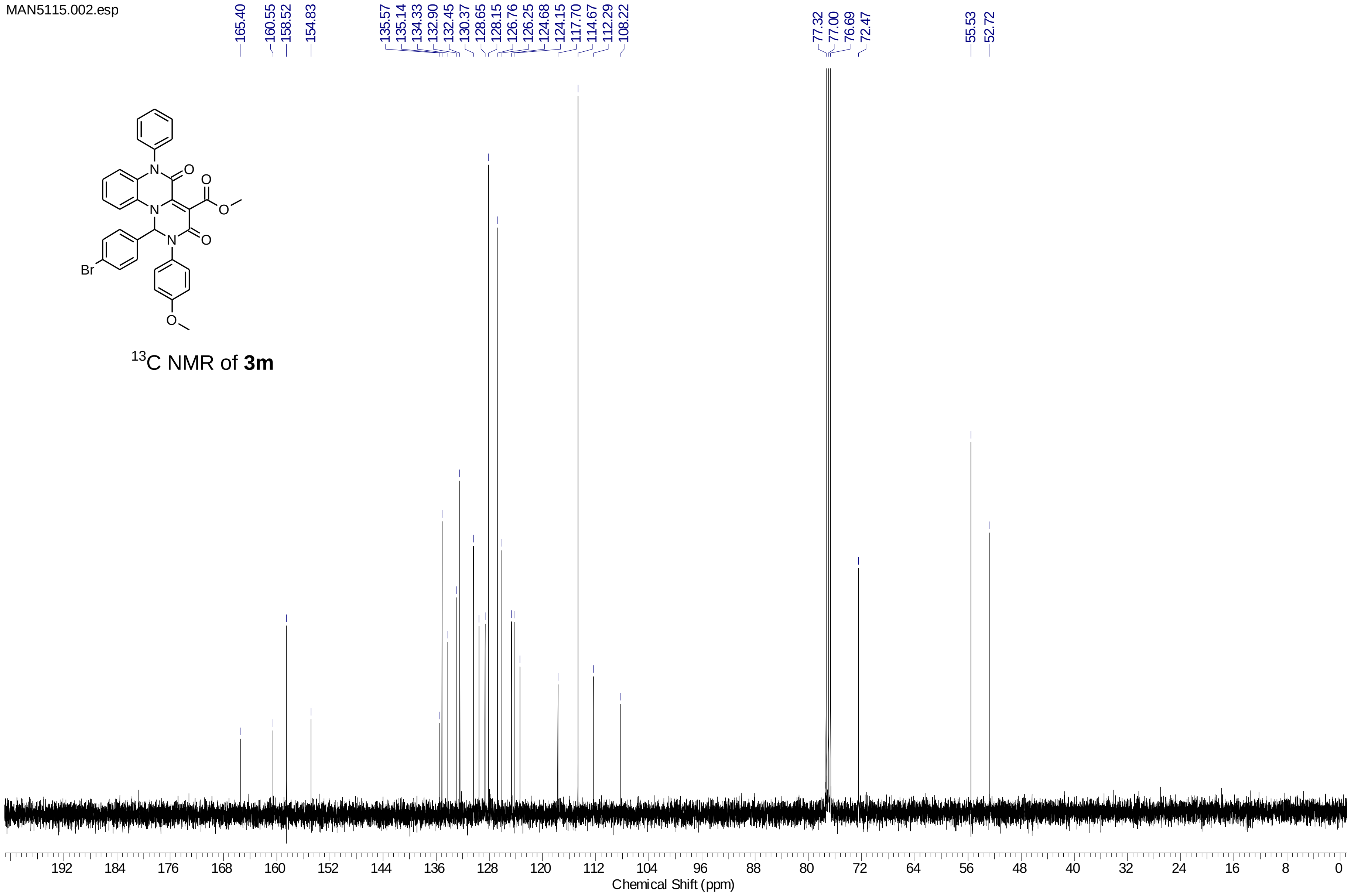
MAN5115.002.esp
165.40
160.55
158.52
154.83
135.57
135.14
134.33
132.90
132.45
130.37
128.65
128.15
126.76
126.25
124.68
124.15
117.70
114.67
112.29
108.22
77.32
77.00
76.69
72.47
55.53
52.72
Br
N
O
192 184 176 168 160 152 144 136 128 120 112 104 96 88 80 72 64 56 48 40 32 24 16 8 0
Chemical Shift (ppm)

^{13}C NMR of **3m**

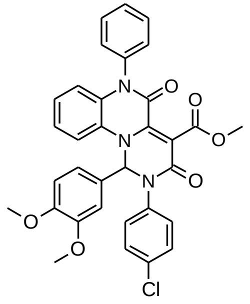

N
O
O
O
N
N
O
O
O
Cl

^{1}H NMR of **3n**

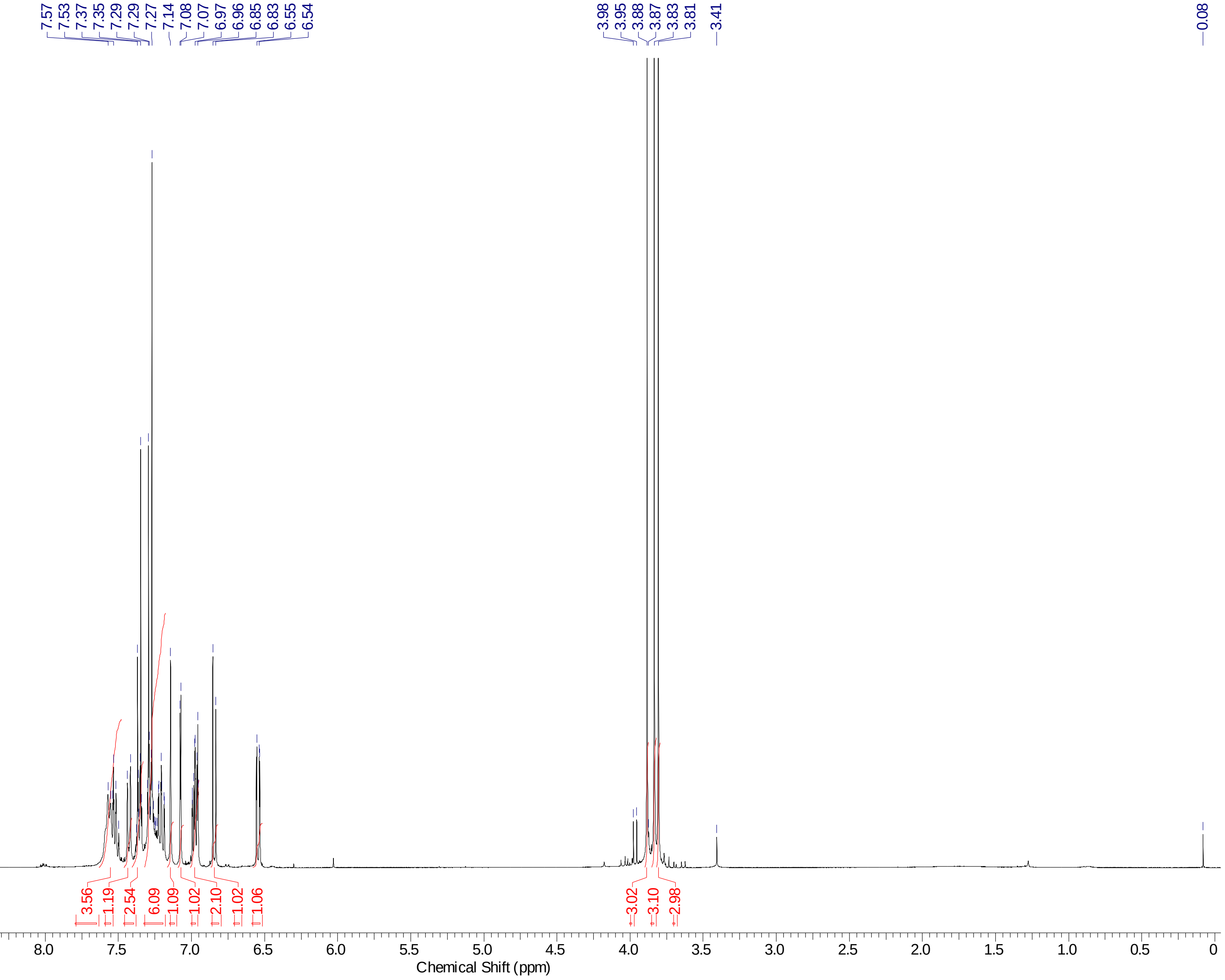

MAN5159.001.esp
7.57
7.53
7.37
7.35
7.29
7.29
7.27
7.14
7.08
7.07
6.97
6.96
6.85
6.83
6.55
6.54
3.98
3.95
3.88
3.87
3.83
3.81
3.41
0.08
3.56
1.19
2.54
6.09
1.09
1.02
2.10
1.02
1.06
3.02
3.10
2.98
9.5
9.0
8.5
8.0
7.5
7.0
6.5
6.0
5.5
5.0
4.5
4.0
3.5
3.0
2.5
2.0
1.5
1.0
0.5
0
Chemical Shift (ppm)

MAN5159.002.esp

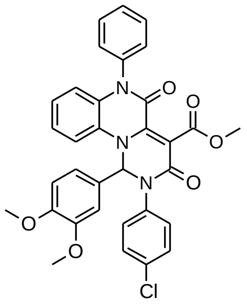

^{13}C NMR of **3n**

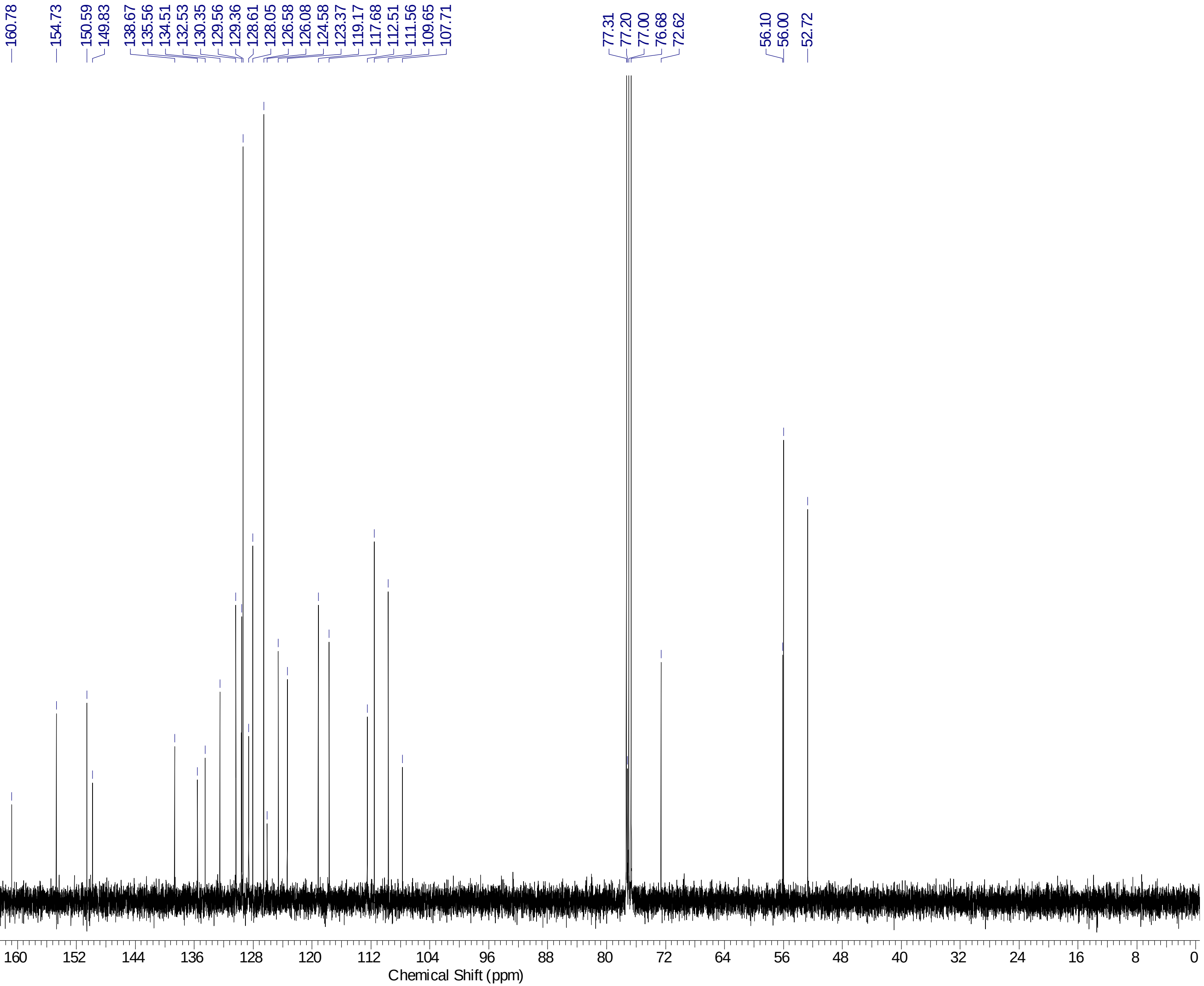

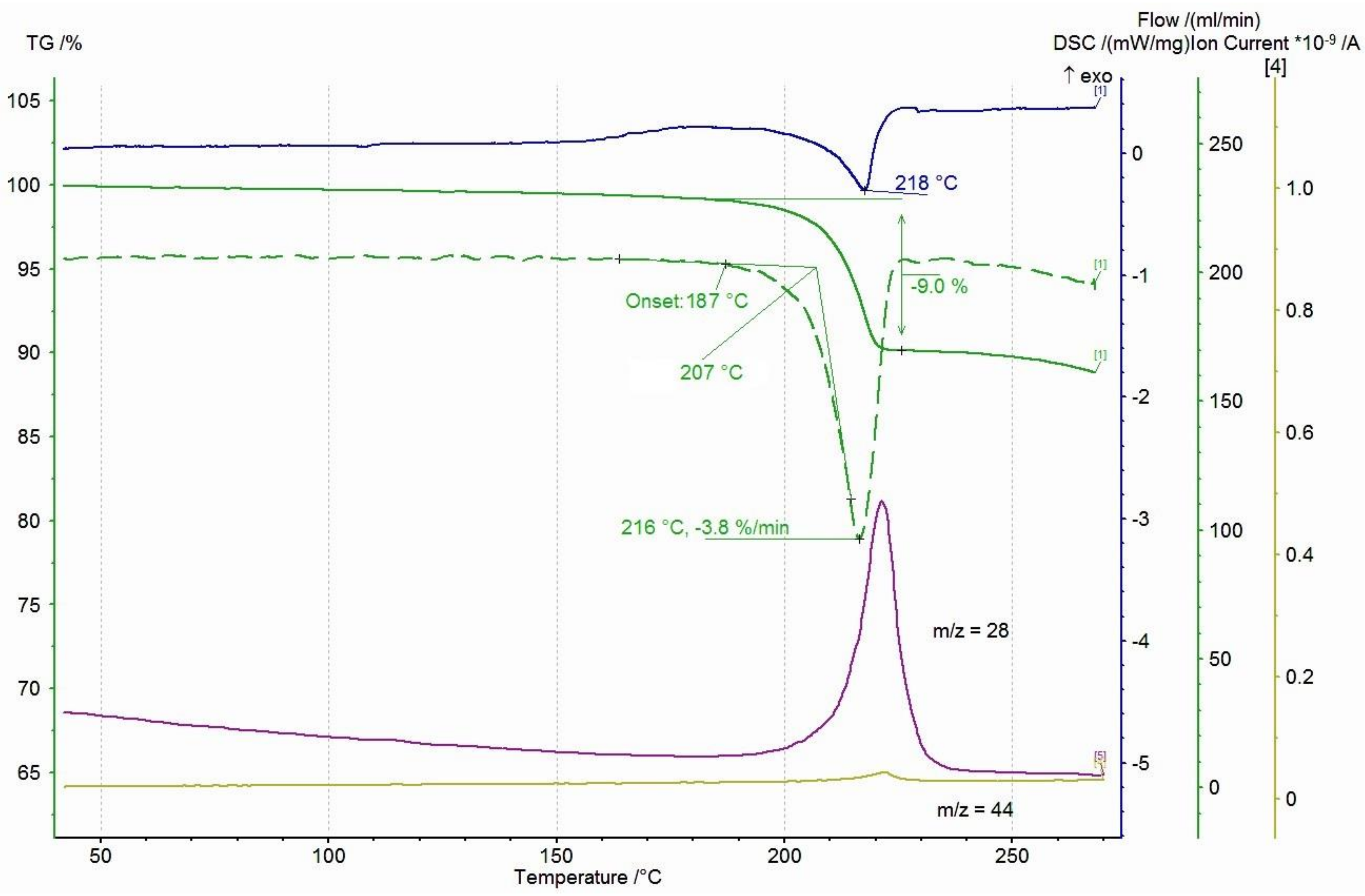

STA plot of thermolysis of PQT **1a**. Blue solid curve: DSC; green solid curve: TG; greed dashed curve: DTG; violet solid curve: MID (*m*/*z* = 28); brown solid curve: MID (*m*/*z* = 44); heating rate: 5 °C/min.

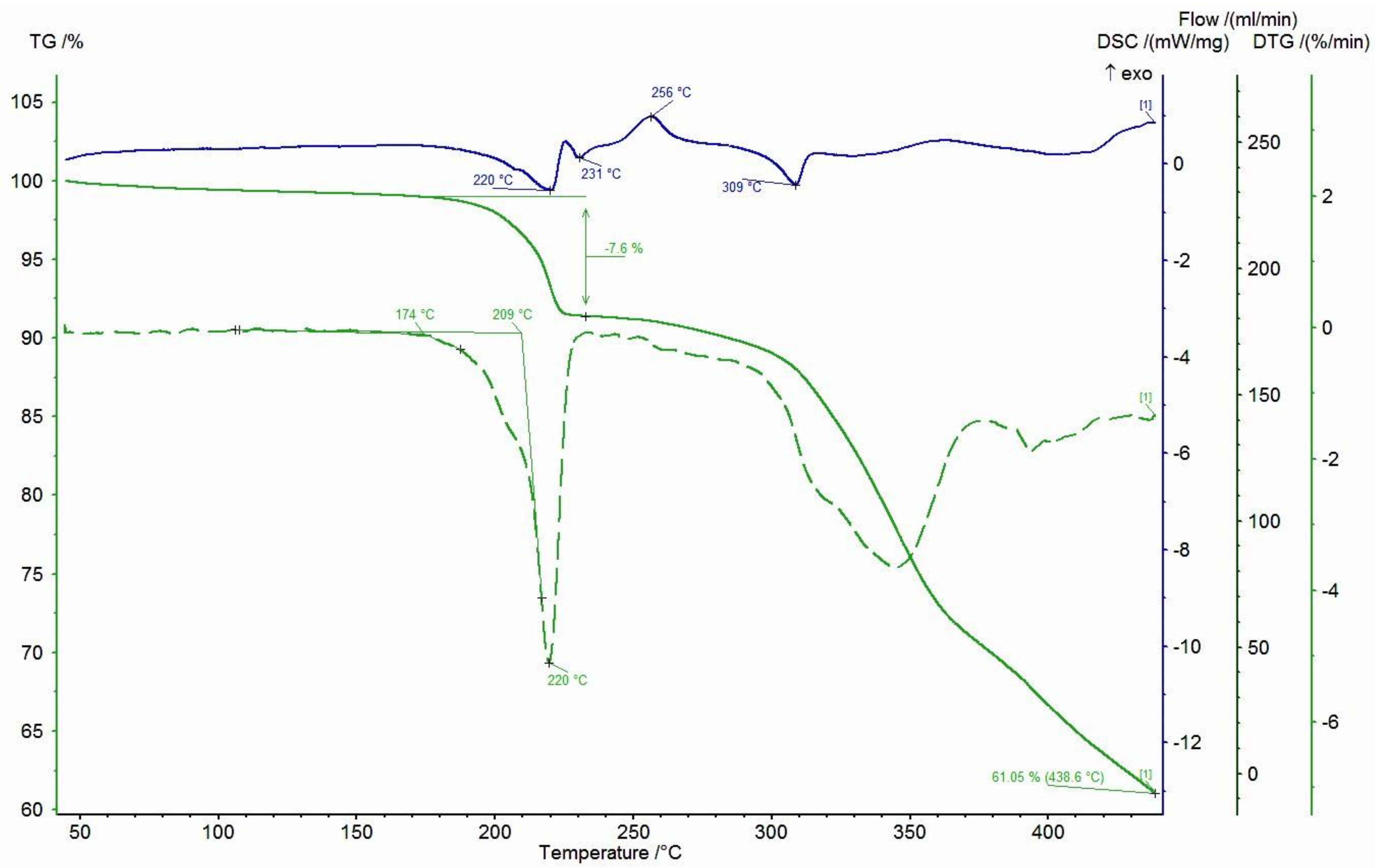

STA plot of thermolysis of PQT **1b**. Blue solid curve: DSC; green solid curve: TG; greed dashed curve: DTG; heating rate: 5 °C/min.

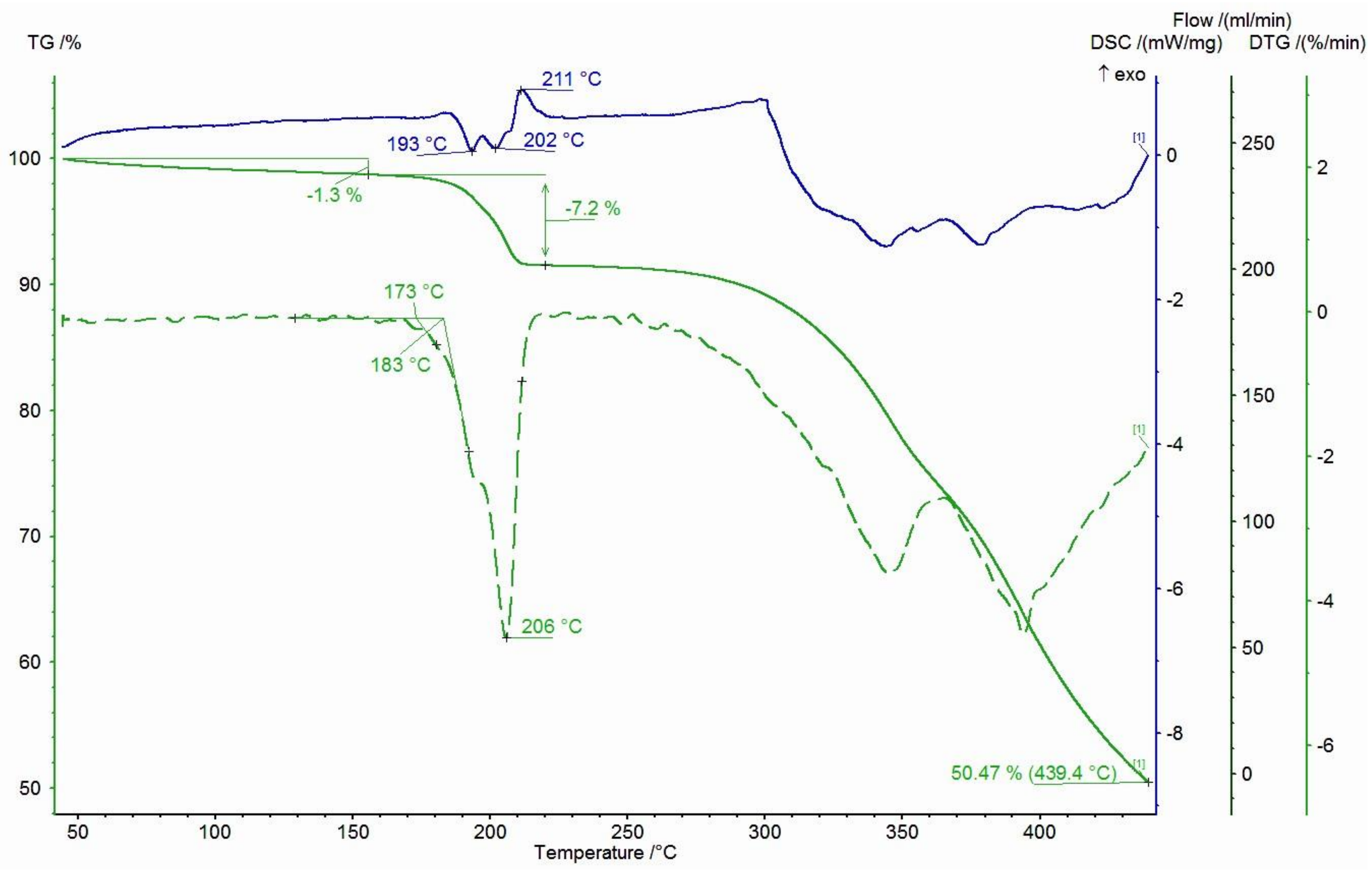

STA plot of thermolysis of PQT **1c**. Blue solid curve: DSC; green solid curve: TG; greed dashed curve: DTG; heating rate: 5 °C/min.

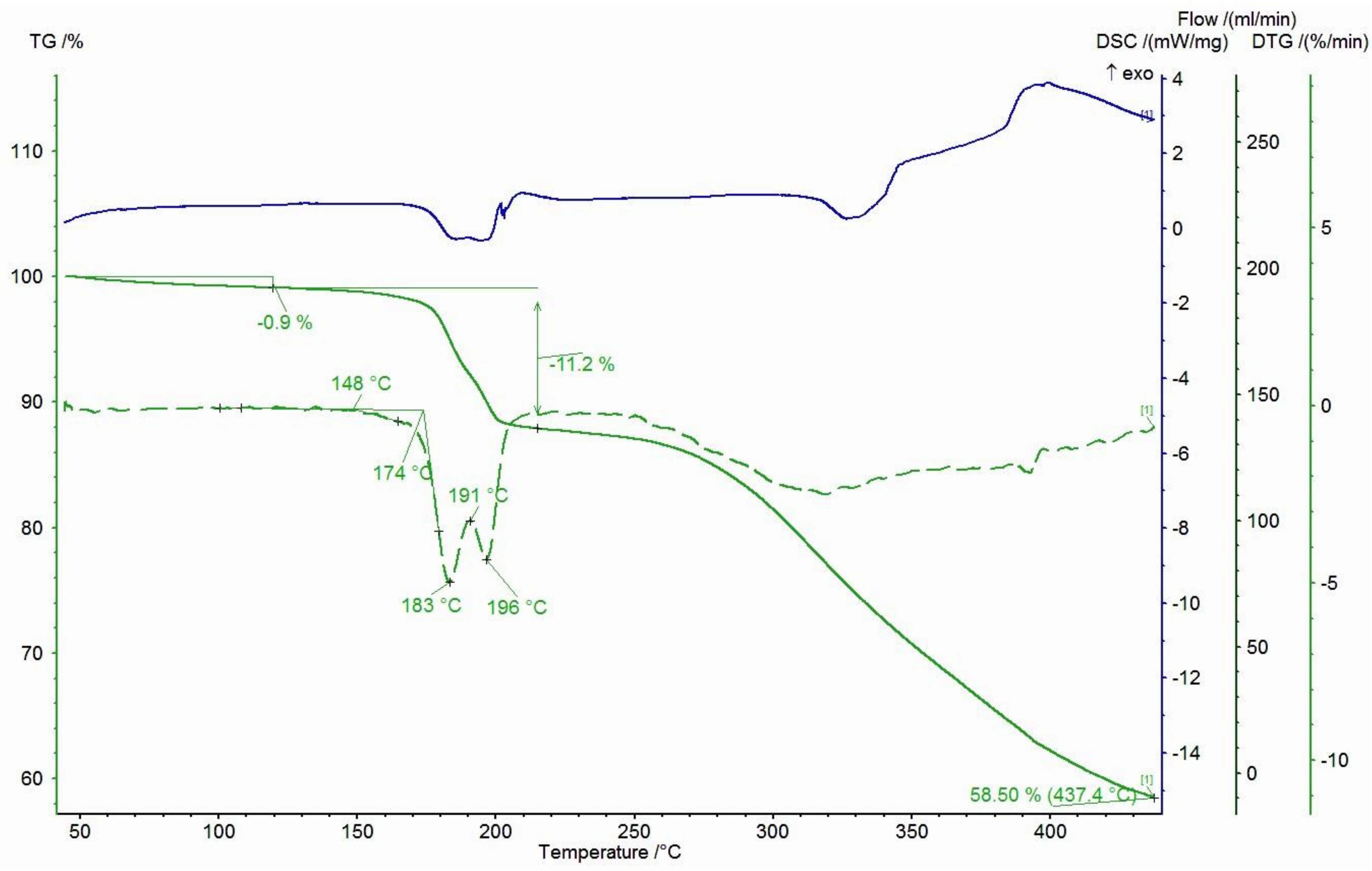

STA plot of thermolysis of PQT **1d**. Blue solid curve: DSC; green solid curve: TG; greed dashed curve: DTG; heating rate: 5 °C/min.

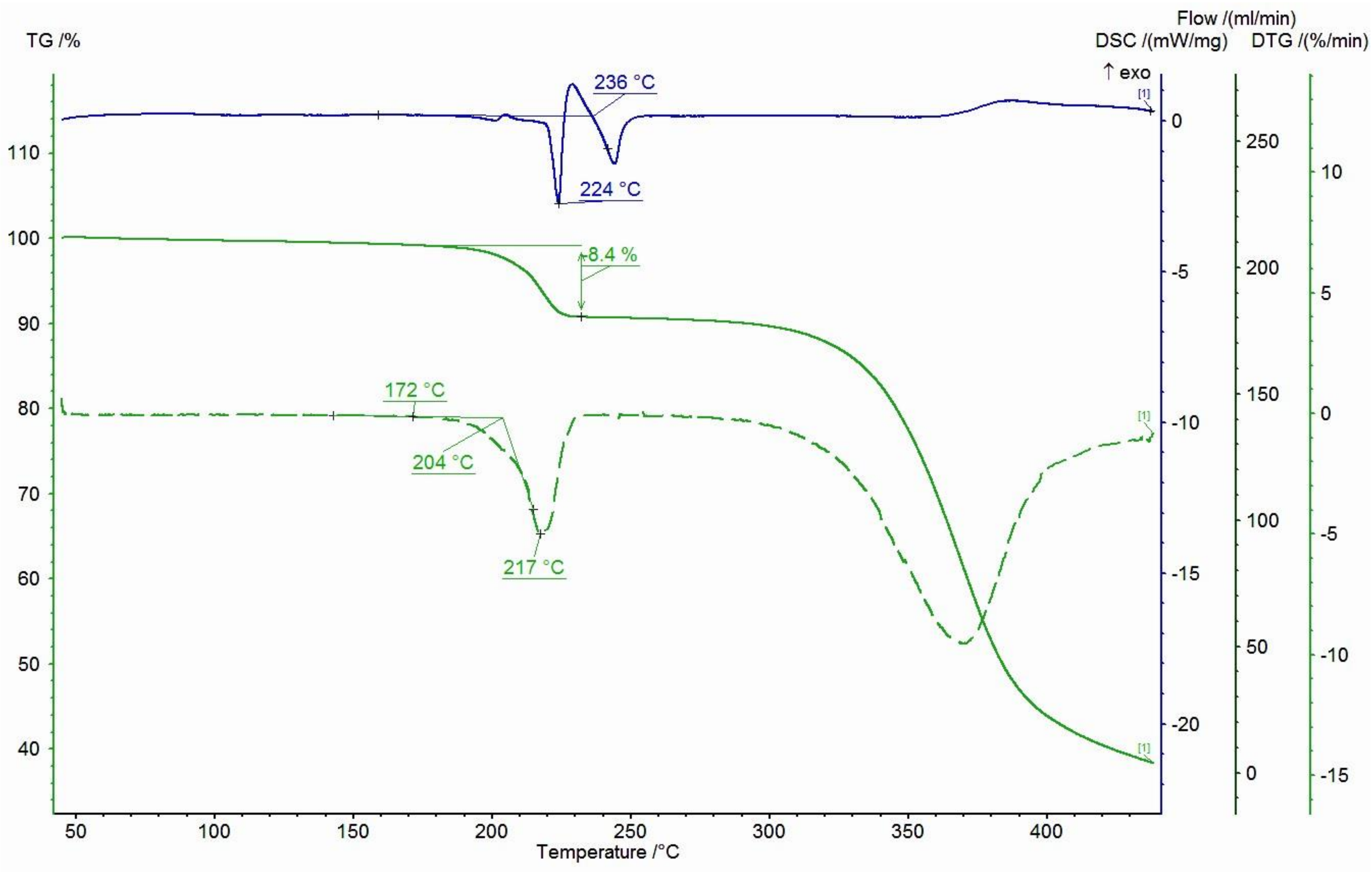

STA plot of thermolysis of PQT **1e**. Blue solid curve: DSC; green solid curve: TG; greed dashed curve: DTG; heating rate: 10 °C/min.

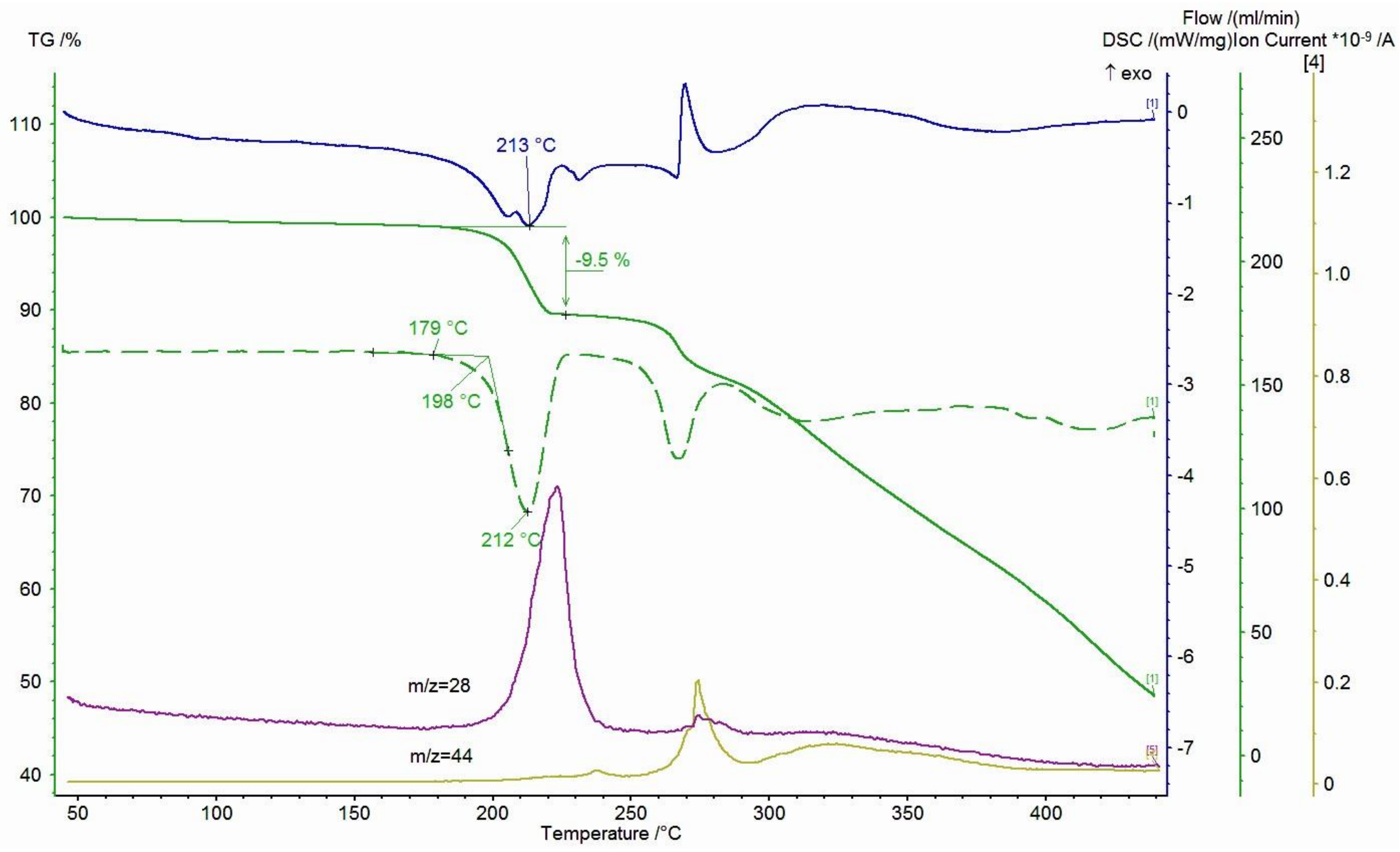

STA plot of thermolysis of PQT **1f**. Blue solid curve: DSC; green solid curve: TG; greed dashed curve: DTG; violet solid curve: MID (*m*/*z* = 28); brown solid curve: MID (*m*/*z* = 44); heating rate: 10 °C/min.

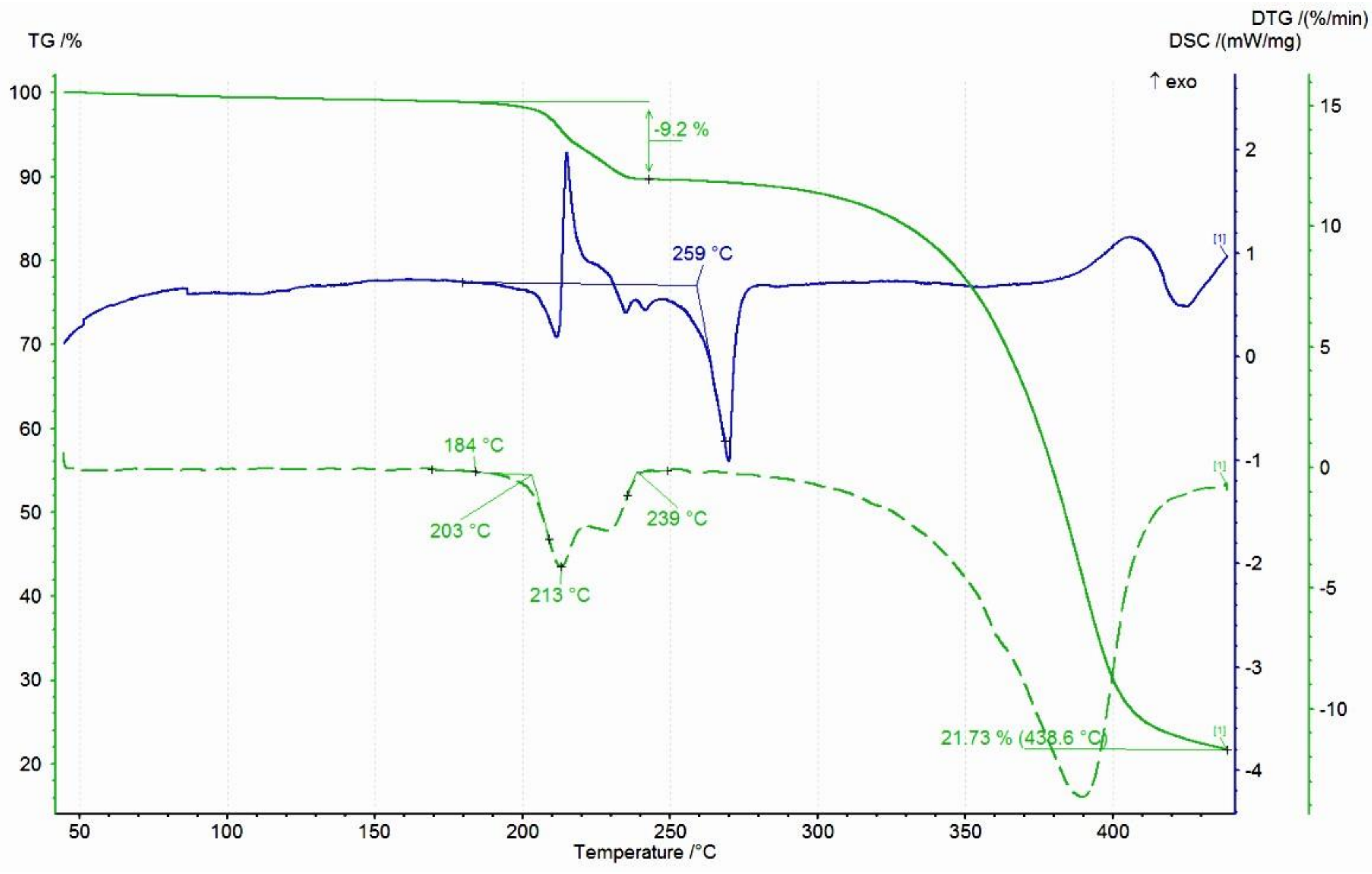

STA plot of thermolysis of PQT **1g**. Blue solid curve: DSC; green solid curve: TG; greed dashed curve: DTG; heating rate: 5 °C/min.

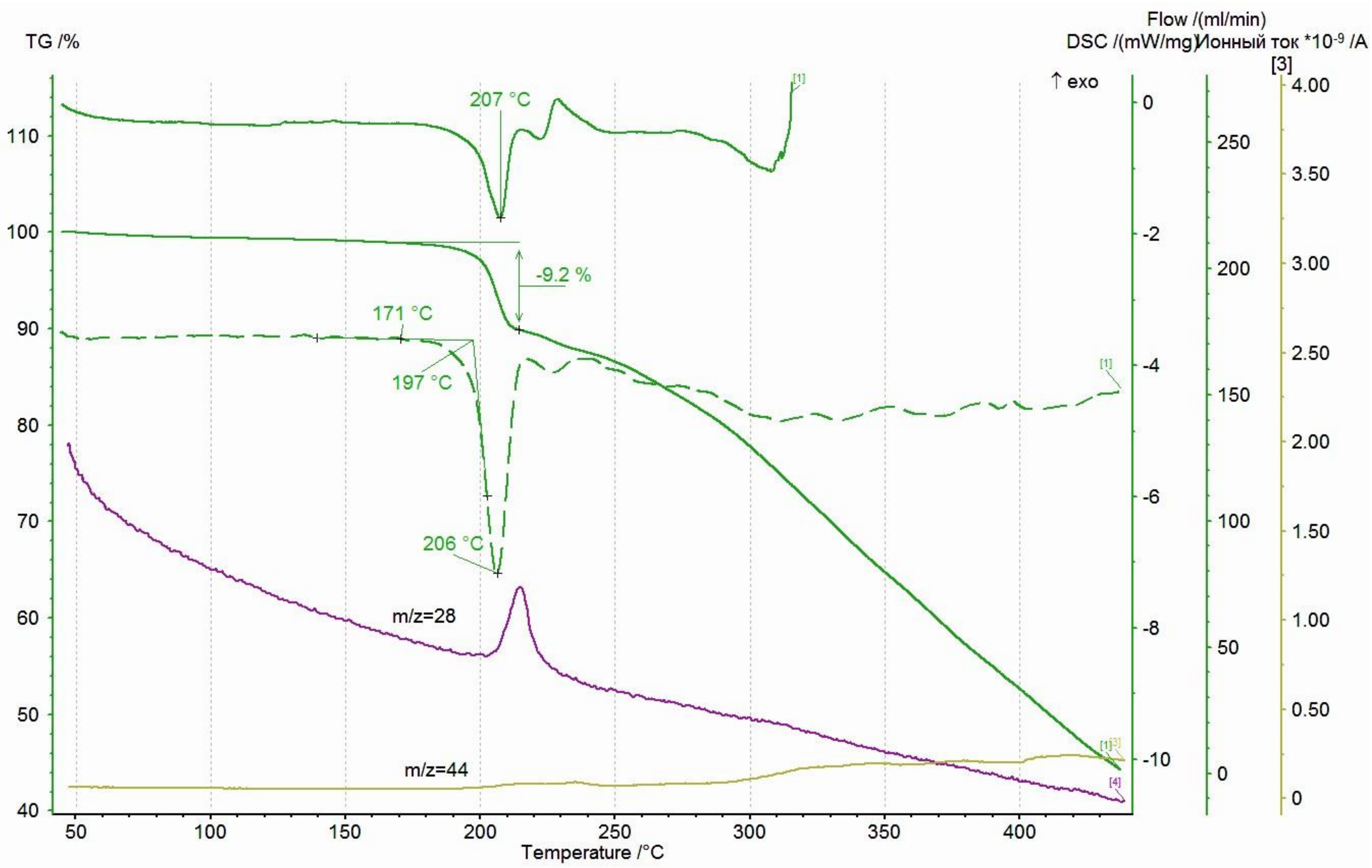

STA plot of thermolysis of PQT **1h**. Green (top) solid curve: DSC; green (second from the top) solid curve: TG; greed dashed curve: DTG; violet solid curve: MID (m/z = 28); brown solid curve: MID (m/z = 44); heating rate: 5 °C/min.

X-ray analysis

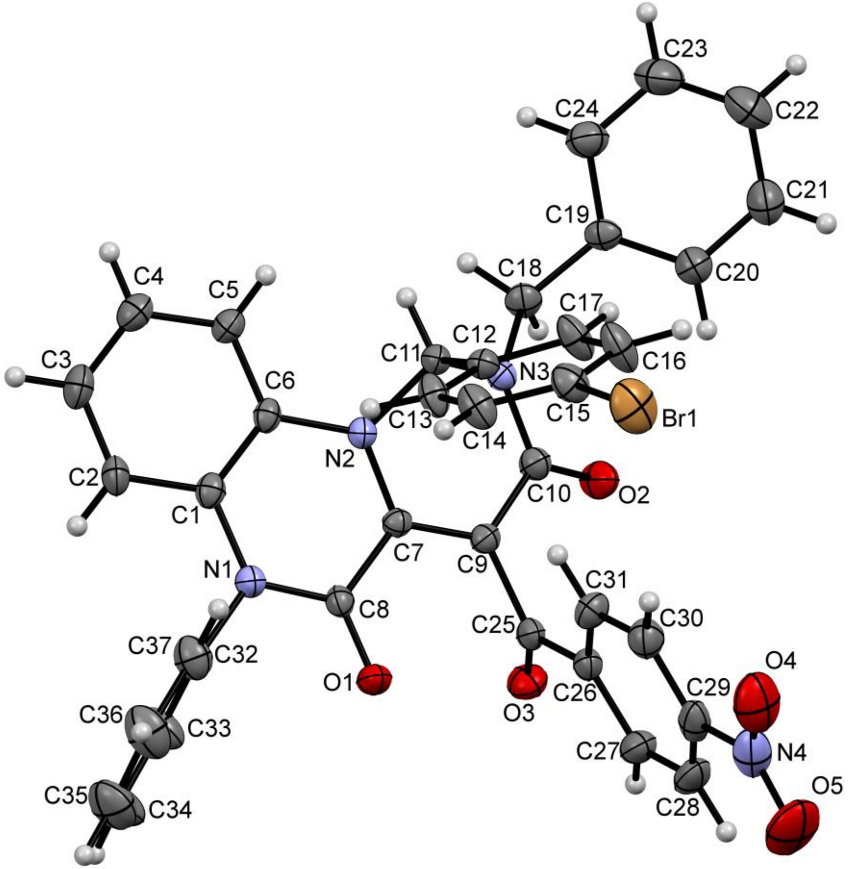

Figure S1. Molecular structure of compound **3g** showing 30% probability amplitude displacement ellipsoids (CCDC 1834011). The solvate molecule is not shown.

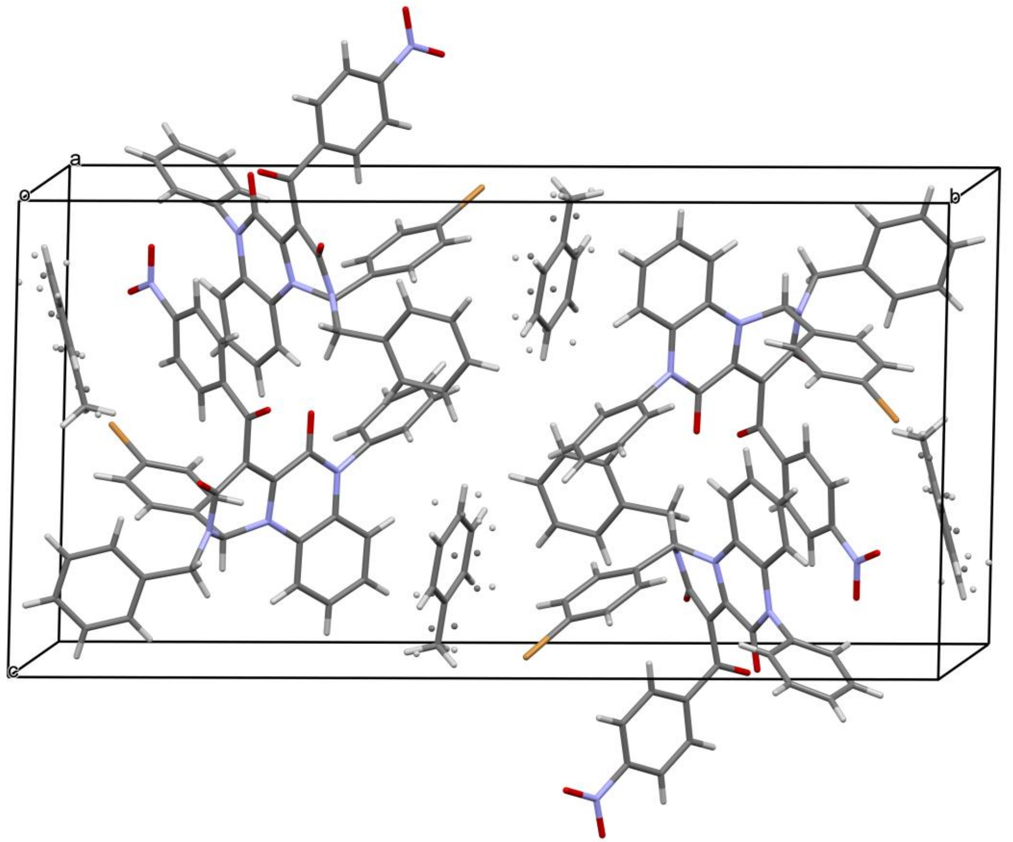

Figure S2. Packing of compound **3g** in crystalline lattice (CCDC 1834011).

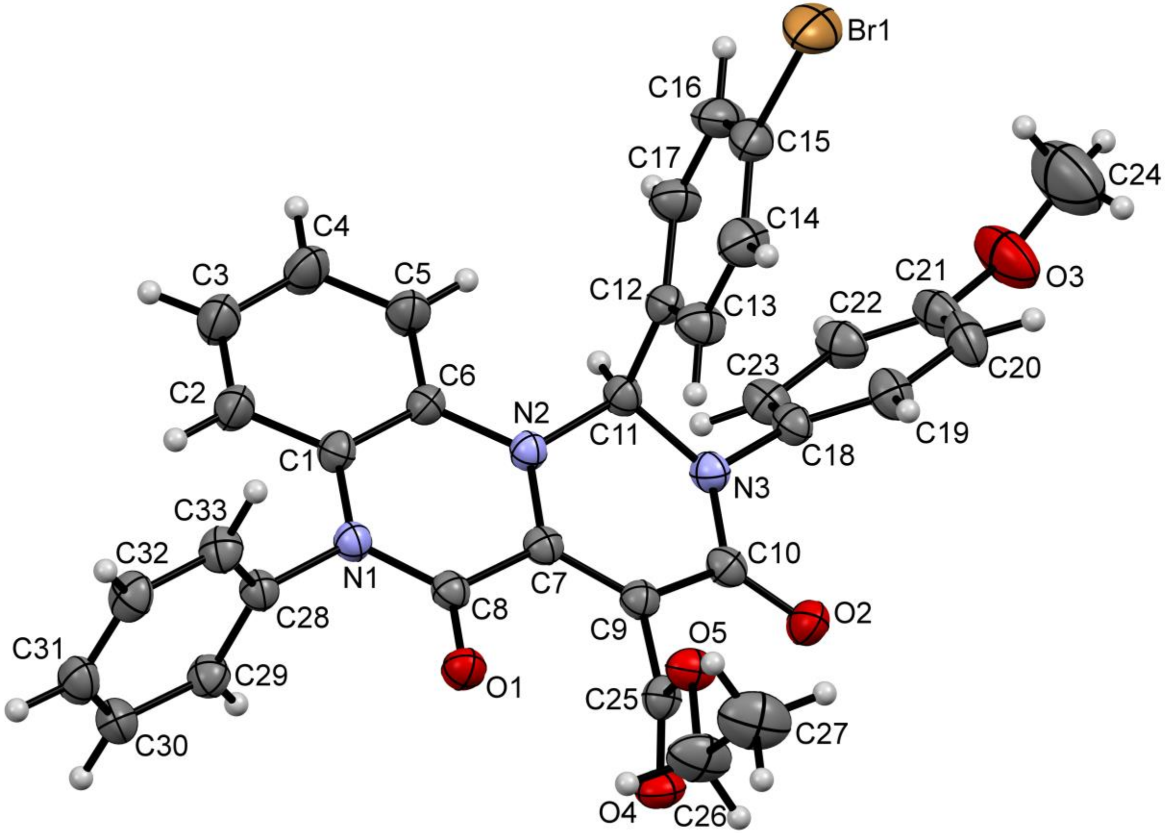

Figure S3. Molecular structure of compound **3j** showing 30% probability amplitude displacement ellipsoids (CCDC 1834012). The solvate molecule is not shown.

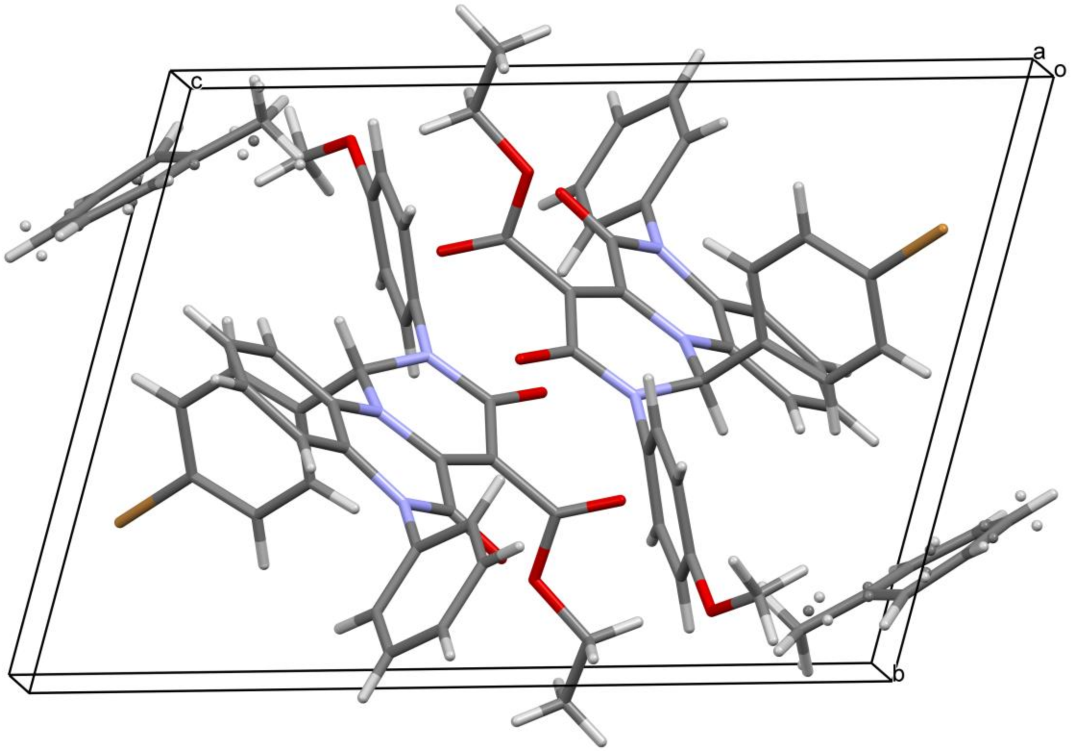

Figure S4. Packing of compound **3j** in crystalline lattice (CCDC 1834012).

Crystal structure determination

The unit cell parameters and the X-ray diffraction intensities were measured on an Xcalibur Ruby diffractometer. The empirical absorption correction was introduced by multi-scan method using SCALE3 ABSPACK algorithm [3]. Using OLEX2 [4], the structures were solved with the olex2.solve program [5] (**3g**) or with the SHELXT program [6] (**3j**) and refined by the full-matrix least-squares method in the anisotropic approximation for all non-hydrogen atoms using the SHELXL [7] program package. Hydrogen atoms were located from the Fourier synthesis of the electron density and refined using a riding model.

Crystal Data of **3g**. $C_{37}H_{25}BrN_4O_5\cdot C_7H_8$, M = 777.65, monoclinic, a = 8.968(2) Å, b = 28.983(7) Å, c = 14.708(4) Å, β = 103.63(3) °, V = 3715.4(15) Å^3, T = 295(2), space group $P2_1/c$, Z = 4, μ(Mo Kα) = 1.159 mm^{-1}. The final refinement parameters: R_1 = 0.0685, wR_2 = 0.1444 [for observed 4284 reflections with $I > 2\sigma(I)$]; R_1 = 0.1614, wR_2 = 0.1926 (for all independent 8777 reflections, R_{int} = 0.0657), S = 1.026.

Crystal Data of **3j**. $C_{33}H_{26}BrN_3O_5\cdot C_7H_8$, M = 716.61, triclinic, a = 10.534(2) Å, b = 11.3633(14) Å, c = 15.2530(17) Å, α = 74.566(10) °, β = 87.497(13) °, γ = 77.768(14) °, V = 1719.8(5) Å^3, T = 295(2), space group P–1, Z = 2, μ(Mo Kα) = 1.245 mm^{-1}. The final refinement parameters: R_1 = 0.0671, wR_2 = 0.1651 [for observed 3882 reflections with $I > 2\sigma(I)$]; R_1 = 0.1459, wR_2 = 0.2241 (for all independent 7940 reflections, R_{int} = 0.0400), S = 1.036.

CCDC 1834011 (for **3g**) and CCDC 1834012 (for **3j**) contain the supplementary crystallographic data for this paper. The data can be obtained free of charge from The Cambridge Crystallographic Data Centre *via* http://www.ccdc.cam.ac.uk.

References:

1. Bozdyreva, K. S.; Smirnova, I. V.; Maslivets, A. N. *Russ. J. Org. Chem.,* **2005,** *41,* 1081–1088. doi:10.1007/s11178-005-0296-6.
2. Mashevskaya, I. V.; Mokrushin, I. G.; Bozdyreva, K. S.; Maslivets, A. N. *Russ. J. Org. Chem.,* **2011,** *47,* 253–257. doi:10.1134/S1070428011020151.
3. CrysAlisPro, Agilent Technologies, Version 1.171.37.33 (release 27-03-2014 CrysAlis171 .NET).
4. Dolomanov, O. V.; Bourhis, L. J.; Gildea, R. J.; Howard, J. A. K.; Puschmann, H. *J. Appl. Cryst.,* **2009,** *42,* 339–341. doi:10.1107/S0021889808042726.
5. Bourhis, L. J.; Dolomanov, O. V.; Gildea, R. J.; Howard, J. A. K.; Puschmann, H. *Acta Cryst*., **2015,** *A71*, 59–75. doi:10.1107/S2053273314022207.
6. Sheldrick, G. M. *Acta Cryst*., **2015**, *A71*, 3–8. doi:10.1107/S2053273314026370.
7. Sheldrick, G. M. *Acta Cryst*., **2015**, *C71*, 3–8. doi:10.1107/S2053229614026540.
